# Hyperbolic band theory

**DOI:** 10.1126/sciadv.abe9170

**Published:** 2021-09-03

**Authors:** Joseph Maciejko, Steven Rayan

**Affiliations:** 1 Department of Physics and Theoretical Physics Institute (TPI), University of Alberta, Edmonton, Alberta T6G 2E1, Canada.; 2 Department of Mathematics and Statistics and Centre for Quantum Topology and Its Applications (quanTA), University of Saskatchewan, Saskatoon, Saskatchewan S7N 5E6, Canada.

## Abstract

The notions of Bloch wave, crystal momentum, and energy bands are commonly regarded as unique features of crystalline materials with commutative translation symmetries. Motivated by the recent realization of hyperbolic lattices in circuit quantum electrodynamics, we exploit ideas from algebraic geometry to construct a hyperbolic generalization of Bloch theory, despite the absence of commutative translation symmetries. For a quantum particle propagating in a hyperbolic lattice potential, we construct a continuous family of eigenstates that acquire Bloch-like phase factors under a discrete but noncommutative group of hyperbolic translations, the Fuchsian group of the lattice. A hyperbolic analog of crystal momentum arises as the set of Aharonov-Bohm phases threading the cycles of a higher-genus Riemann surface associated with this group. This crystal momentum lives in a higher-dimensional Brillouin zone torus, the Jacobian of the Riemann surface, over which a discrete set of continuous energy bands can be computed.

## Teaser

The band theory of solids—including Bloch waves, crystal momentum, and energy bands—is generalized to hyperbolic lattices.

## INTRODUCTION

The concept of Bloch wave is a cornerstone of modern physics. Introduced by Felix Bloch in 1928 to describe the quantum-mechanical propagation of electrons in crystalline solids (*1*), this phenomenon applies generally to the propagation of waves of any kind in periodic media, including atomic matter waves in optical lattices, light in photonic crystals, and sound in acoustic metamaterials. The key condition for the existence of a Bloch wave is periodicity of the underlying medium—specifically, that the latter be composed of identical unit cells that are repeated under elementary translations. A Bloch wave traveling through such a medium is not itself a periodic function but acquires predictable phase shifts under those elementary translations. The phase shifts, in turn, define the crystal momentum ***k*** of the wave and its associated reciprocal space. (While our discussion is applicable to wave phenomena in general, for concreteness we will use the language of quantum condensed matter physics and a units convention familiar in that field, whereas the reduced Planck’s constant ħ is set to one, and the terms “momentum” and “wave vector,” and “energy” and “frequency,” are used interchangeably.) Because the allowed translations are discrete, the crystal momentum is itself a periodic variable, and an irreducible set of inequivalent crystal momenta is given by the (first) Brillouin zone. This basic fact is the foundation upon which the edifice of band theory is built (*2*). Energy levels are organized into energy bands, a discrete set {*E_n_*(***k***)} of continuous functions of ***k*** over the Brillouin zone. In *d* spatial dimensions, the latter is topologically equivalent to a *d*-dimensional torus. Our focus is on *d* = 2 spatial dimensions, where this topological space is an ordinary torus, homeomorphic to the surface of a doughnut. The nontrivial topology of the Brillouin zone, stemming from the periodicity of crystalline lattices, is ultimately responsible for the topological revolution in condensed matter physics, initiated by Haldane’s discovery of the Chern insulator (*3*) and firmly established through the development of a comprehensive topological band theory (*4*).

The absence of periodicity, that is, of a discrete translation symmetry in the system’s underlying Hamiltonian, substantially complicates the theoretical study of wave propagation. In a limited number of cases, band theory may still serve as a starting point. Localized or weak deviations from strict periodicity can often be successfully modeled as perturbations of a periodic Hamiltonian, as in standard theories of impurity states or randomized impurity scattering in crystals (*2*). Incommensurate modulated phases and quasicrystals, while strongly aperiodic, can be described as projections of ordinary periodic lattices in higher dimensions (*5*). Key aspects of wave propagation in such media, such as the existence of sharp Bragg peaks in x-ray diffraction, can thus be understood via analogous projections of a correspondingly higher-dimensional reciprocal space (*6*). In spite of these cases, the central tenets of band theory—crystal momentum, the toroidal Brillouin zone, and sharply defined energy bands—are expected to fundamentally break down in generic aperiodic media.

Recently, an example of a new class of synthetic aperiodic structures has been engineered using the technology of circuit quantum electrodynamics (*7*). The structure is an ordered but aperiodic network of microwave resonators that, from the point of view of wave propagation, can be described effectively as a regular heptagonal tessellation of the hyperbolic plane. Such tessellations, also known as hyperbolic tilings, were studied by Coxeter (*8*) and popularized through M. C. Escher’s now famous “Circle Limit” woodcuts (*9*). As with an ordinary two-dimensional (2D) crystal such as graphene, whose geometry corresponds to a regular tiling of the Euclidean plane, a hyperbolic tiling consists of repeated unit cells that are all geometrically identical but allows for patterns impossible in Euclidean space, such as a tiling by regular heptagons (*7*). That such a tiling is only possible in hyperbolic space follows from the fact that the latter is endowed with a uniform negative curvature. As a result, the sum of the interior angles of an *n*-sided polygon is strictly less than (*n* − 2)π, and repeated unit cells are identical in the sense of non-Euclidean geometry. Put somewhat differently, using the phrase “geometrically identical” to describe the unit cells depends crucially upon comparing them under the lens of a particular choice of metric, the hyperbolic or Poincaré metric. As such, hyperbolic tilings are also qualitatively distinct from quasicrystalline ones, which tile the Euclidean plane (albeit aperiodically) and in which the unit cells are identical under the standard Euclidean metric. In the experiments of Kollár *et al*. (*7*), negative curvature is simulated by artificially engineering the couplings between the resonators such that resonators that appear closer together from a Euclidean vantage point—near the circular edge of Escher’s artwork (*9*), metaphorically speaking—are coupled with the same strength as resonators near the center of the device, which appear further apart.

Spurred by this experimental breakthrough, recent theoretical studies have explored the propagation of matter waves on hyperbolic lattices. Using graph theory and numerical diagonalization, Kollár *et al*. (*10*) obtained general mathematical results concerning the existence of extended degeneracies and gaps in the spectrum of tight-binding Hamiltonians on a variety of discrete hyperbolic lattices. Boettcher *et al*. (*11*) developed a hyperbolic analog of the effective-mass approximation in solid-state physics, showing that such tight-binding Hamiltonians reduce in the long-distance limit to the hyperbolic Laplacian—the Laplace-Beltrami operator associated with the Poincaré metric on the hyperbolic plane—and proposing the synthetic structures of Kollár *et al*. (*7*) as a new platform for the simulation of quantum field theory in curved space. Topological quantum phenomena in hyperbolic lattices were explored using real-space numerical diagonalization by Yu *et al.* (*12*). Notwithstanding these notable advances, quoting Kollár *et al*. (*7*), “no hyperbolic equivalent of Bloch theory currently exists, and there is no known general procedure for calculating band structures in either the nearly-free-electron or tight-binding limits.” The authors have thus concluded that explicit spectra can only be obtained using numerical diagonalization, “a brute-force method which yields a list of eigenvectors and eigenvalues, but no classification of eigenstates by a momentum quantum number” (*7*).

In this work, we present the first hyperbolic generalization of Bloch theory. We show that aperiodic Hamiltonians with the symmetry of a particular class of hyperbolic tilings can be described by such a generalization, which we dub hyperbolic band theory. Despite the absence of a commutative, discrete translation group, we show that a hyperbolic crystal momentum ***k*** can be suitably defined, but lives in a vector space of dimension higher than two. There exists a corresponding hyperbolic Brillouin zone that is topologically equivalent to a higher-dimensional, compact torus. A hyperbolic bandstructure {*E_n_*(***k***)}, a discrete set of continuous functions of ***k*** on this higher-dimensional Brillouin zone, can be defined and explicitly computed.

**Fig. 1.**
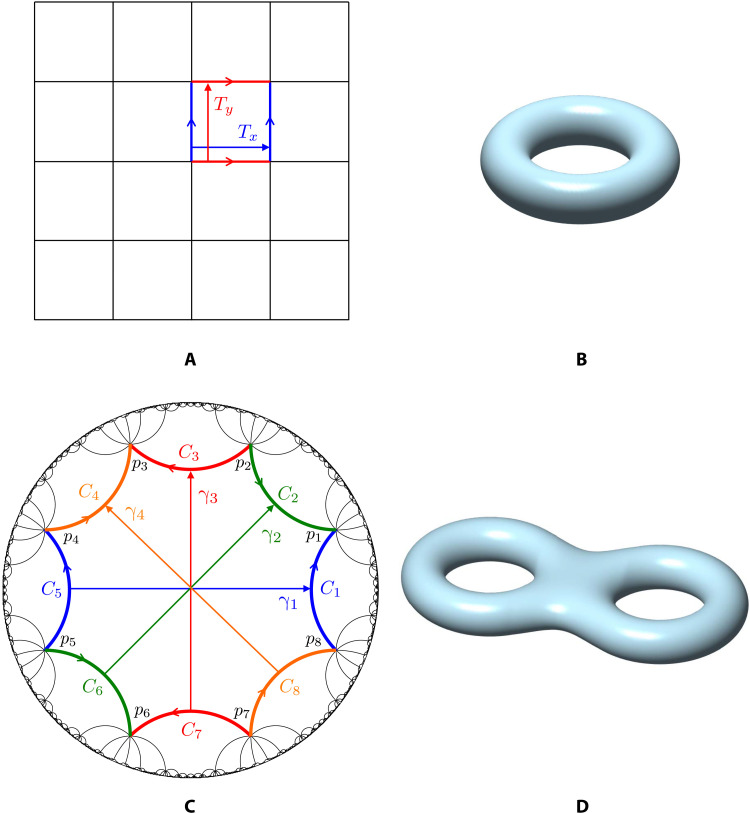
Euclidean versus hyperbolic lattices. For the Euclidean lattice (**A**), the unit translations, denoted as *T_x_* and *T_y_* here, identify pairwise the four sides of the unit cell, which gives the ordinary torus (**B**). On the hyperbolic lattice (**C**), Fuchsian group transformations γ_1_, γ_2_, γ_3_, and γ_4_ identify pairwise the eight sides of the hyperbolic unit cell, which gives the genus-2 surface (**D**). In both (A) and (C), the identifications preserve the orientations of the sides, which are indicated by arrows.

The higher-dimensional torus that is the hyperbolic Brillouin zone is related to the tessellation of the 2D hyperbolic plane through a particular construction commonly studied in the field of algebraic geometry in mathematics. It emerges naturally from our setup that the torus always has even dimension 2*g*, where *g* is the genus, or number of holes, of a compact Riemann surface. The torus and the Riemann surface are related in a precise mathematical way: The Brillouin zone is exactly the Jacobian (*13*) of the surface. The Riemann surface is itself a minimal representation of the original configuration space, arising after quotienting the hyperbolic plane by a noncommutative translation group Γ, called a Fuchsian group, which amounts to identifying pairs of edges of a 4*g*-sided fundamental cell (see Fig. 1C).

From the point of view of the presence of Riemann surfaces, our hyperbolic band theory is simultaneously a higher-genus band theory. In comparison, ordinary band theory is a genus-one theory, where the now standard 2D torus arises as the quotient of the Euclidean plane by a commutative, discrete group of lattice translations, where the lattice is determined by a (4 × 1)–sided fundamental cell. The torus serves both as a minimal representation of the configuration space and as reduced momentum space. Viewed from algebraic geometry, the real space torus is a genus-one Riemann surface known commonly as an elliptic curve and the momentum space torus is the Jacobian of the elliptic curve. It is a classical fact from algebraic geometry that an elliptic curve and its Jacobian are isomorphic, not only topologically but also as complex manifolds. The equivalence between them is given by the Abel-Jacobi map (*13*), which can be thought of as a geometric Fourier transform. Because of their identical geometry, one can pass easily back and forth between the two tori, blurring the lines between position space and momentum space when convenient. In our hyperbolic band theory, the Riemann surface and the Jacobian no longer share the same topology, nor even the same dimension. Still, the passage between them is given by a higher-dimensional Abel-Jacobi map, which can be approximated numerically as required. The realization of the role of algebraic geometry in what has been, until now, a squarely topological theory of materials anticipates a plethora of new constructions and algebro-geometric invariants for describing and classifying quantum material structures.

## RESULTS AND DISCUSSION

### Euclidean lattices and Bloch phases

We begin by reviewing Bloch theory for Euclidean lattices. In the absence of a periodic potential, the propagation of electrons on the 2D Euclidean plane $E≅{\mathbb{R}}^{2}$ is described by the usual free-particle Hamiltonian *H*_0_ = ***p***^2^/2*m*, where *m* is the electron mass and ***p*** = − *i*∇ is the momentum operator. The continuous translation invariance of *H*_0_ is expressed mathematically by the fact that it commutes with the operator *T*_***a***_ = *e*^−*i****p***·***a***^ for translations of E by an arbitrary vector ***a***. Likewise, its continuous *SO*(*2*) symmetry under planar rotations corresponds to the fact that *H*_0_ commutes with *e*^−*i*θ*L_z_*^, the rotation operator through angle θ, with *L_z_* = − *i∂*/∂θ the angular momentum operator. Together, translations and rotations form the special Euclidean group *SE*(2) of rigid motions of E. In the presence of a periodic potential *V*(*x*, *y*), the Hamiltonian is augmented as *H* = *H*_0_ + *V* and is now only invariant under a discrete subgroup *G* ⊂ *SE*(2). We shall take the potential to have the symmetry of a square lattice (Fig. 1A), with the lattice constant set to unity. As Bloch’s theorem is a consequence of the periodicity of *H* exclusively (*2*), we will ignore point-group operations and take *G* to be the Abelian group of discrete translations on the lattice. The latter is isomorphic as a group to ℤ × ℤ and is generated by the unit translations in the *x* and *y* directions, respectively. Bloch’s theorem states that eigenstates of *H* enjoy the property ψ(*x* + 1, *y*) = *e^ik_x_^*ψ(*x*, *y*), ψ(*x*, *y* + 1) = *e^ik_y_^*ψ(*x*, *y*). Because *k_x_* and *k_y_* appear as phase factors, they are determined up to integer multiples of 2π. It follows that ***k*** = (*k_x_*, *k_y_*) lives in the first Brillouin zone, which is the 2-torus given by the product of two circles, each of unit radius.

Eigenfunctions of *H* satisfying the Bloch condition can be explicitly constructed as follows. One solves the Schrödinger equation in a reference unit cell 𝒟, say [0,1] × [0,1], with the twisted, periodic boundary conditions ψ(1, *y*) = *e^ik_x_^*ψ(0, *y*), ψ(*x*,1) = *e^ik_y_^*ψ(*x*,0), and identical conditions on ∂*_x_*ψ and ∂*_y_*ψ, obtained by taking derivatives of the earlier Bloch condition. Because the unit cell is a compact region and the Hamiltonian is self-adjoint on the space of twice-differentiable, square-integrable functions on 𝒟 with such boundary conditions, one obtains a discrete set of real eigenvalues *E_n_*(***k***) for *H* on 𝒟. Since *H* is the same in every unit cell, the corresponding solution on the entire Euclidean plane E is simply obtained by translating the solution in 𝒟 in a manner that respects the Bloch condition. The solution at position ***r*** = (*x*, *y*) in a unit cell displaced from 𝒟 by the lattice translation ***R*** = (*R_x_*, *R_y_*) ∈ ℤ^2^ is given in terms of the solution in 𝒟 by ψ(***r***) = *e*^*i****k***·***R***^ψ(***r*** − ***R***). This function obeys the Schrödinger equation and the Bloch condition everywhere, and the function and its derivatives are manifestly continuous.

To generalize the ideas at play to the hyperbolic case, it will be useful to reinterpret this manner of constructing Bloch waves for *H* as follows. In reducing the Schrödinger problem on E to its solution on a single unit cell 𝒟, we replace E with its quotient by *G*. This action produces a 2-torus: $E/G≅{\mathbb{R}}^{2}/{\mathbb{Z}}^{2}≅T^{2}$ (Fig. 1B). The Bloch phase factors *e^ik_x_^* and *e^ik_y_^* can then be interpreted as Aharonov-Bohm phases produced by fluxes, *k_x_* = ∮*_C_x__A* and *k_y_* = ∮*_C_y__A*, which thread the two noncontractible cycles *C_x_*,*C_y_* of this torus, where *A* is a flat connection on the torus. Alternatively, each Bloch phase factor can be viewed as a *U*(1)-representation of the fundamental group of the torus, π_1_(*T*^2^), which is generated by the homotopy classes *C_x_* and *C_y_* and obeys the presentation $C_{x}C_{y}C_{x}^{-1}C_{y}^{-1}=1$ (*14*). The representation $\chi (C_{x,y})=\chi {\left(C_{x,y}^{-1}\right)}^{*}=e^{ik_{x,y}}\in U(1)$ manifestly obeys this presentation. Note that π_1_(*T*^2^) ≅ ℤ^2^ is isomorphic to *G*. Thus, we recover the usual point of view according to which the Bloch phase factors form a *U*(1)-representation of the discrete translation group.

What may be overlooked is that, strictly speaking, the construction above involves two homeomorphic 2-tori. The first, which we denote Σ, is the one obtained by taking the real configuration space E and quotienting by the symmetry group of the lattice. The second, which we shall call the Jacobian of Σ and denote Jac(Σ), is obtained from collecting the Bloch phase factors into a topological space, which naturally has the topology of a 2-torus. We take as a definition that Jac(Σ) is the set of all representations of π_1_(Σ) into *U*(1), although, classically, there are several distinct-appearing yet equivalent ways to define the Jacobian (*13*). It is also crucial to observe that these two spaces are not simply topological tori but rather complex manifolds. The torus Σ was constructed from orthogonal unit translations, which correspond to the basis vectors 1 on the real axis and *i* on the imaginary one. Their ratio τ = *i*/1 = *i* is the value of a parameter in the complex upper half-plane that determines a particular elliptic curve, which is a compact Riemann surface of genus 1. The Riemann surface structure is extra geometric information on top of the topological structure of the torus. At the same time, the choice of τ determines a particular elliptic curve structure on Jac(Σ), which we can take to be identical to that of Σ. In general, there is no canonical choice of identification between an elliptic curve and its Jacobian. Any such identification depends upon a choice of base point, which is the base point for the Abel-Jacobi map, and changes of base point are simply translations of the lattice. Another interesting observation is that both Σ and Jac(Σ) are algebraic groups, as elliptic curves come equipped with an Abelian group law, the existence of which has tremendous implications for number theory and cryptography [e.g., (*15*)]. On the Jacobian side, the group structure manifests in the addition of crystal momenta, ***k*** + ***k***′, modulo the reciprocal lattice.

### Hyperbolic lattices and automorphic Bloch phases

We now turn to hyperbolic lattices. By analogy with the Euclidean case, the Hamiltonian of an electron propagating freely in the 2D hyperbolic plane ℍ should be invariant under the group of rigid motions of this space, which is isomorphic to the projective special unitary group *PSU*(1,1) ≅ *SU*(1,1)/{ ± *I*}. [The key aspects of hyperbolic geometry we will be needing here, such as the Poincaré disk model, the compactification of the hyperbolic octagon, and the hyperbolic Laplacian, are reviewed in a language accessible to scientists in (*16*).] We will be mostly working with the Poincaré disk model of the hyperbolic plane, in which ℍ corresponds to the interior of the complex unit disk ∣*z*∣ < 1, with the Poincaré metric given by the line element *ds*^2^ = 4(1 − ∣*z*∣^2^)^−2^(*dx*^2^ + *dy*^2^), with *z* = *x* + *iy*. The Poincaré disk model also underlies the effectively non-Euclidean geometry of the engineered structures in (*7*). Elements γ of the symmetry group act on a point *z* ∈ ℍ by Möbius transformations: *z* → γ(*z*) = (α*z* + β)/(β^*^z + α^*^), where ∣α∣^2^ − ∣β∣^2^ = 1. Because the Euclidean free-particle Hamiltonian *H*_0_ is proportional to the Euclidean Laplacian ∇^2^, its natural generalization to the hyperbolic case is *H*_0_ = − Δ, where

$$\Delta =\frac{1}{4}{\left(1-{|z|}^{2}\right)}^{2}(\frac{\partial^{2}}{\partial x^{2}}+\frac{\partial^{2}}{\partial y^{2}})$$ (1)

is the Laplace-Beltrami operator on the Poincaré disk ℍ, which we will refer to as the “hyperbolic Laplacian.” One then explicitly checks that Δ commutes with Möbius transformations.

To introduce a hyperbolic lattice, we consider a potential *V*(*x*, *y*) with the symmetry of a {4*g*, 4*g*} hyperbolic tiling with *g* ≥ 2. The unit cell of such tilings, which are impossible in Euclidean space, is a hyperbolic 4*g*-gon, 4*g* of which meet at each vertex of the lattice. We first outline the key steps of our construction for general tilings of this type and later proceed with detailed calculations for a specific example: the Poincaré regular octagonal {8,8} tiling (*g* = 2) illustrated in Fig. 1C.

The full Hamiltonian *H* = *H*_0_ + *V* is now not invariant under continuous *PSU*(1,1) transformations, but rather under the discrete Fuchsian subgroup Γ determined by the tiling. While non-Abelian in general, this group behaves as a hyperbolic analog of a discrete translation group: It acts properly discontinuously on the hyperbolic plane ℍ, meaning that its repeated action on a single fundamental region or reference unit cell 𝒟 in ℍ tiles all of ℍ with geometrically identical copies of 𝒟, with neither gaps nor overlaps (*17*). We will focus on the case where Γ is co-compact and strictly hyperbolic, in which case the unit cell 𝒟 is compact and has finite area under the Poincaré metric. For the {4*g*, 4*g*} tiling, 𝒟 is a hyperbolic 4*g*-gon, meaning a polygon whose 4*g* sides are geodesic segments under the metric. The uniformization theorem—an important result appearing in algebraic geometry, differential geometry, and number theory—states that the quotient ℍ/Γ is a smooth, compact Riemann surface Σ*_g_* of genus *g* ≥ 2 (*18*). Topologically, this surface originates from 2*g* pairwise identifications of the sides of 𝒟 under the action of Γ. Such a surface has 2*g* noncontractible cycles, corresponding to homotopy classes *a*_1_, *b*_1_, …, *a_g_*, *b_g_* through a common base point *p*_0_ ∈ Σ*_g_* and through which 2*g* Aharonov-Bohm fluxes $k_{a}^{\left(1\right)},k_{b}^{\left(1\right)},\ldots ,k_{a}^{\left(g\right)},k_{b}^{\left(g\right)}\in [0,2\pi )$ can be threaded, each of which can again be interpreted as the integral of a flat connection around the corresponding cycle. What also persists is that the 2*g* phase factors $e^{ik_{a}^{\left(1\right)}},e^{ik_{b}^{\left(1\right)}},\ldots ,e^{ik_{a}^{\left(g\right)}},e^{ik_{b}^{\left(g\right)}}$ form a *U*(1)-representation χ of the fundamental group π_1_(Σ*_g_*) of Σ*_g_*, which is generated by the homotopy classes of Σ*_g_*, as seen in the relation $a_{1}b_{1}a_{1}^{-1}b_{1}^{-1}\cdots a_{g}b_{g}a_{g}^{-1}b_{g}^{-1}=1$ that defines π_1_(Σ*_g_*). We define χ in terms of the Aharonov-Bohm fluxes by $\chi (a_{i})=\chi {\left(a_{i}^{-1}\right)}^{*}=e^{ik_{a}^{\left(i\right)}}$, $\chi (b_{i})=\chi {\left(b_{i}^{-1}\right)}^{*}=e^{ik_{b}^{\left(i\right)}}$, *i* = 1, …, *g*. In analogy with the Euclidean case, the Fuchsian group Γ is isomorphic to π_1_(Σ*_g_*). Isomorphic subgroups Γ ⊂ *PSU*(1,1) that generate the same {4*g*, 4*g*} hyperbolic tiling are the analog of distinct choices of basis vectors for the same periodic lattice in the Euclidean case.

In this geometric picture, we again have two complex manifolds, although they are no longer isomorphic—not as complex manifolds and not even topologically. One is the Riemann surface Σ*_g_*, which is a minimal domain for the real configuration space. As in the Euclidean case, there is a particular complex manifold structure on Σ*_g_* that is inherited from the quotient by Γ and, hence, from the particular choice of tessellation. The other manifold is Jac(Σ*_g_*), the Jacobian of Σ*_g_*, which parametrizes distinct *U*(1)-representations χ of π_1_(Σ*_g_*). The manifold Σ*_g_* is 2D just as in the Euclidean case, although it is no longer homeomorphic to a 2-torus. On the other hand, Jac(Σ*_g_*) is 2*g*-dimensional and is homeomorphic to the 2*g*-torus *T*^2*g*^ = (*S*^1^)^2*g*^. Yet another difference is that while Jac(Σ*_g_*) remains a group under addition of phases, Σ*_g_* does not admit a group law.

From these observations, we propose that, despite the absence of an Abelian translation group, the choice of a {4*g*, 4*g*} hyperbolic lattice induces naturally a notion of crystal momentum: a 2*g*-dimensional hyperbolic crystal momentum, $k=(k_{a}^{\left(1\right)},k_{b}^{\left(1\right)},\ldots ,k_{a}^{\left(g\right)},k_{b}^{\left(g\right)})\in T^{2g}≅\text{Jac}\;(\Sigma_{g})$. In other words, we propose that Jac(Σ*_g_*) plays the role of a hyperbolic Brillouin zone. By analogy with the Euclidean case described earlier, the notion of hyperbolic crystal momentum can be used to construct eigenfunctions ψ of *H* starting from a single reference unit cell 𝒟. For *z* = *x* + *iy* in the Poincaré disk, we generalize the Bloch condition to

ψ(γ(z))=χ(γ)ψ(z) (2)

where γ ∈ Γ acts by Möbius transformations and where χ is the map discussed earlier. Appearing as early as works of Poincaré, functions obeying the condition (Eq. 2) are known as automorphic functions with factor of automorphy χ (*19*, *20*) and can be seen as hyperbolic analogs of periodic functions. [We use a convention standard in the number-theory literature; from the point of view of representation theory, a more natural but physically equivalent convention is ψ(γ^−1^(*z*)) = χ(γ)ψ(*z*), which simply amounts to replacing χ(γ) by χ(γ)^−1^.] The factor of automorphy here is the simplest possible type—that of weight 0, also known as a multiplier system. More generally, one may consider factors of automorphy that depend holomorphically on *z*, that is, weight-*k* factors of automorphy $\hat{\chi }(\gamma ,z)=\chi (\gamma ){\left(\mathrm{cz}+d\right)}^{k}$ for some real numbers *c* and *d*, and where χ : Γ → *U*(1) and *z* ∈ ℍ. We consider only unitary automorphic factors in our Bloch condition, as a direct generalization of the Euclidean situation.

By assumption, the potential *V* itself is an automorphic function with trivial automorphy factor, *V*(γ(*z*)) = *V*(*z*). Accordingly, we shall refer to such a potential as an automorphic potential. Again, we solve the Schrödinger equation

(−Δ+V)ψ=Eψ (3)

on the single reference unit cell 𝒟, with the boundary conditions specified by Eq. 2. By analogy with the Euclidean or genus-1 case, there are now 2*g* linearly independent boundary conditions to apply, corresponding to the 2*g* generators of π_1_(Σ*_g_*). In practice, one requires an explicit representation of those generators as *PSU*(1,1) matrices. The potential *V* does not involve derivatives and is thus trivially self-adjoint. With the boundary conditions (Eq. 2), the hyperbolic Laplacian Δ can be shown to be self-adjoint on 𝒟 as well (*21*). Because the region is compact, we obtain a discrete set of real eigenvalues {*E_n_*(***k***)} for each value of the hyperbolic crystal momentum ***k***. Since *H* is the same in every unit cell, i.e., it is invariant under the action of Γ, the solution on 𝒟 can be extended to the entire Poincaré disk ℍ by Γ-translating it in a manner that respects the generalized Bloch condition (2). In other words, the solution in any fundamental domain 𝒟′ ⊂ ℍ, which is necessarily the image of 𝒟 under the action of a particular element γ ∈ Γ, is given by ψ(*z*) = χ(γ)ψ(γ^−1^(*z*)), where *z* ∈ 𝒟′ and γ^−1^(*z*) ∈ 𝒟. This construction ensures that, as in the Euclidean case, ψ obeys the Schrödinger equation (Eq. 3) and the generalized Bloch condition (2) everywhere, and ψ and its derivatives are continuous in the entire Poincaré disk. With these observations in hand, we have the desired identifications: Jac(Σ*_g_*) is our hyperbolic momentum space, and we may describe each factor of automorphy χ as a hyperbolic Bloch phase.

### Particle-wave duality and the Abel-Jacobi map

The geometry emerging from our construction is a pair of complex manifolds, Σ*_g_* and Jac(Σ*_g_*). In the Euclidean case, these manifolds manifest as a pair of essentially indistinguishable elliptic curves. One can ask whether we retain a direct passage from one to the other in the hyperbolic, or *g* ≥ 2, case.

To this end, we shall very briefly review the complex manifold structure of the Riemann surface Σ*_g_*. The surface Σ*_g_* is covered by overlapping open patches *U*, each of which is homeomorphic under a map ψ*_U_* to an open set of the complex plane ℂ. This allows us to assign to a point *p* ∈ *U* a coordinate, which is the corresponding complex number ψ*_U_*(*p*). When *p* is in the intersection of two open patches *U* and *V*, we can translate from one coordinate system to another via $\psi_{V}\circ \psi_{U}^{-1}$. The fact that Σ*_g_* is a Riemann surface implies that there exists such an atlas of coordinate charts in which each and every composition $\psi_{V}\circ \psi_{U}^{-1}$ is holomorphic as a map between two open sets in ℂ, meaning that the composite functions satisfy the standard Cauchy-Riemann equations of complex analysis. [The complex manifold structure of Fuchsian quotients such as Σ*_g_* is further discussed in (*22*).]

The fact that Σ*_g_* looks locally like an open set in ℂ means that it is also possible to discuss complex-valued holomorphic functions *f* on Σ*_g_*; locally, they are holomorphic functions from *U* to ℂ. Moreover, we have an associated notion of holomorphic one-form: These are the one-forms on Σ*_g_* that can be written locally as θ = *fdz*, where *f* is a holomorphic function on Σ*_g_*.

A well-known result in algebraic geometry that follows from the Riemann-Roch theorem and Serre duality [e.g., (*23*, *24*)] is that the global holomorphic one-forms on Σ*_g_* constitute a vector space of complex dimension *g*. In other words, there are *g*-many global, linearly independent, holomorphic one-forms θ_1_, …, θ*_g_* on the Riemann surface. This is an algebraic interpretation of the genus that complements the topological one: Rather than counting the number of holes, we think of *g* as counting the number of independent one-forms—a fact consistent with the reality that there is no global holomorphic one-form on the Riemann sphere other than θ = 0.

Now, recall that we chose 2*g* cycles with a common base point *p*_0_ via which we defined Aharonov-Bohm fluxes, leading to *U*(1)-representations χ of π_1_(Σ*_g_*). These cycles provide a basis for the first homology group of the surface. At this point, we will replace these cycles with a symplectic basis, which is a collection of loops *a_i_*, *b_i_*, *i* = 1, …, *g*, such that *a_i_* and *b_i_* intersect in exactly one point and all other intersections are empty. At the same time, we choose a basis θ_1_, …, θ*_g_* of holomorphic one-forms in such a way that they are “dual” to the *a* loops, meaning that ∮*_a_i__*θ*_j_* = δ*_ij_*. The remaining integrals, which form *g*-many *g*-tuples (∮_*b*_1__θ*_j_*, …, ∮*_b_g__*θ*_j_*), produce a nondegenerate *g* × *g* matrix Ω, the period matrix of Σ*_g_*. The full rank of Ω follows from the Riemann bilinear relations (*13*). Hence, the columns are a basis for ℂ*^g^*, giving us a lattice structure on the underlying ℝ^2*g*^, known as the period lattice. Let us denote this lattice by Λ. The quotient ℝ^2*g*^/Λ is precisely Jac(Σ*_g_*). The matrix Ω can be shown to be always symmetric with positive-definite imaginary part. The space of all such matrices is called the Siegel upper half-space. Note that in the *g* = 1 or elliptic curve case, the period matrix is 1 × 1—it is precisely the modular parameter τ in the upper half-plane.

Now, let *p* be any point in Σ*_g_* and let *c_p_* be a continuous path from *p*_0_ to *p*, where *p*_0_ is a base point (not necessarily the one we chose earlier). We can define a map *a* : Σ*_g_* → Jac(Σ*_g_*) by setting

$$a(p)=(\int_{c_{p}}\theta_{1},\ldots ,\int_{c_{p}}\theta_{g})\;\text{mod}\;\Lambda$$ (4)

Here, the integral yields a vector in ℂ*^g^* ≅ ℝ^2*g*^. We then translate the output to the fundamental unit cell in ℂ*^g^* ≅ ℝ^2*g*^ of the lattice Λ, thus producing a point in Jac(Σ*_g_*)—equivalently, a crystal momentum ***k***. It is readily apparent that the map is independent of both the specific base point, as well as the chosen path to *p*. Changing the path perturbs the calculation by an integral over a cycle, which can be written in the basis (*a_i_*, *b_i_*), and so the difference that we pick up is precisely an element of Λ. This difference is killed by the quotient. Changing the base point simply translates the torus. Last, when we take *p* = *p*_0_, we are integrating only over cycles, which again are killed by the quotient, and so, *a*(*p*_0_) = ***k*** = 0 is the identity in the Jacobian as a group.

The map defined here is the Abel-Jacobi map. As it maps a 1D space (over ℂ) to a *g*-dimensional space, it is only an isomorphism in the genus-1 case, where it provides the familiar particle-wave duality of Euclidean quantum mechanics and, hence, conventional band theory. Intuitively, the line integrals in Eq. 4 can be interpreted as the set of topologically distinct contributions to the geometric phase accumulated under adiabatic motion of a quantum particle from a reference point *p*_0_ to a given point *p* inside the unit cell. In the Euclidean case, the unit cell is geometrically flat, and the two contributions to the geometric phase are linear functions of two linearly independent displacements, producing an isomorphism between real and momentum spaces. Apart from the obvious dimensional differences inherent in the hyperbolic case, the nontrivial negative curvature of the unit cell required by the Gauss-Bonnet theorem renders such a linear mapping impossible.

To counter the difference in dimension between the configuration and momentum spaces for *g* ≥ 2, we can ask about the effect of applying the map in Eq. 4 to *g*-tuples of points from Σ*_g_*, by defining $a(p_{1},\ldots ,p_{g})=\sum_{i=1}^{g}a(p_{i})$. One immediate observation is that the order of the *g* inputs has no effect on the output, and so, the map is well-defined on the symmetric product of the Riemann surface with itself *g* times (rather than simply the Cartesian product). It is a classical fact from algebraic geometry, e.g., (*25*), that this map *a* from the *g*-fold symmetric product of Σ*_g_* to Jac(Σ*_g_*) is almost an isomorphism of complex manifolds. The map is only birational, which means that a certain submanifold of the symmetric product must be “blown down” to recover Jac(Σ*_g_*). This submanifold is an example of a “high-symmetry” region, related to the so-called theta divisor in Jac(Σ*_g_*) (*13*). While involving some technical aspects of algebraic geometry, this construction exhibits the Jacobian as a particular complex manifold arising from the data of our Riemann surface in a direct way, providing an algebraic particle-wave duality that exists in spite of dimensional and curvature differences.

The aforementioned high-symmetry region is worthy of further investigation, as it suggests the existence of a special set of points in the hyperbolic unit cell whose physical relevance is not yet appreciated. The map further suggests that an ideal Liouville-Arnold–type phase space for this physical system in which the configuration space and momentum space have equal dimension might be given by a fibration of Jacobian tori over a *g*-dimensional complex space associated to Σ*_g_*. We leave the formalization of this dynamical system to forthcoming work. In the meantime, we now proceed with a concrete example of our hyperbolic band theory in genus *g* = 2.

### The Bolza lattice

Having outlined the key ideas of our general theory, we now apply it to the simplest hyperbolic analog of the Euclidean square lattice: the regular octagonal {8,8} tiling depicted in Fig. 1C. This tiling is generated by the action of a Fuchsian group Γ on a reference unit cell 𝒟, which can be taken to be the regular hyperbolic octagon centered at the origin *z* = 0 of the Poincaré disk, that is, the region bounded by the colored geodesic segments *C*_1_, …, *C*_8_ in Fig. 1C (see also the Supplementary Materials). We define the hyperbolic Bloch factor χ(γ) in Eq. 2 by its action on the Fuchsian group generators γ*_j_*: $\chi (\gamma_{j})=\chi {\left(\gamma_{j}^{-1}\right)}^{*}=e^{ik_{j}}$, *j* = 1, …,4, writing the hyperbolic crystal momentum as ***k*** = (*k*_1_, *k*_2_, *k*_3_, *k*_4_) ∈ Jac(Σ_2_). In this case, the underlying topology of the hyperbolic Brillouin zone Jac(Σ_2_) is a 4D torus *T*^4^. Because we require χ to be a representation of Γ, we further define χ(γ*_i_*γ*_j_*) = χ(γ*_i_*)χ(γ*_j_*) for any *i*, *j* = 1, …,4. Since Γ is finitely generated by the γ*_j_*, this is sufficient to define χ(γ) for any γ ∈ Γ. Combining the definition of the hyperbolic Bloch factor with the automorphic Bloch condition (2) and the pairwise identifications imposed by Γ, the four boundary conditions we impose when solving the hyperbolic Bloch problem (Eq. 3) on the hyperbolic octagon 𝒟 become ψ(*C_j_*) = *e^ik_j_^*ψ(*C*_*j* + 4_), *j* = 1, …,4.

In ordinary band theory, the simplest problem that illustrates many salient features of generic bandstructures, including zone folding and symmetry-protected or accidental degeneracies, is the empty-lattice approximation (*2*). In this approximation, the potential is taken to be constant, and thus necessarily periodic; without loss of generality, one can further choose *V* = 0. As we then have *H* = *H*_0_ = − ∇^2^/2*m*, the problem thus reduces to finding the eigenvalues and eigenfunctions of the Euclidean Laplacian with Bloch (twisted) boundary conditions. One easily finds $E_{n}(k)=\frac{1}{2m}{\left(k+2\pi n\right)}^{2}$ and ψ_***nk***_(***r***) ∝ *e*^*i*(***k*** + 2π***n***)·***r***^, with ***k*** ∈ *T*^2^ as the crystal momentum and ***n*** = (*n_x_*, *n_y_*) ∈ ℤ^2^ as a discrete band index.

In the hyperbolic case, we wish to find the eigenvalues *E* and eigenfunctions ψ of the hyperbolic Laplacian −Δ on the hyperbolic octagon 𝒟 with the twisted boundary conditions mentioned earlier. At the origin of the hyperbolic Brillouin zone, ***k*** = 0, those boundary conditions reduce to the condition that the solutions be strictly automorphic, ψ(γ(*z*)) = ψ(*z*), the case usually considered in mathematics (*26*).

While exact analytical solutions for the eigenfunctions and eigenenergies are unavailable, this problem can be studied numerically. Motivated by questions in the theory of quantum chaos, approximate eigenenergies and eigenfunctions of the hyperbolic Laplacian on hyperbolic octagons with strictly automorphic boundary conditions were first obtained by Aurich and Steiner (*27*) using the finite element method. Subsequent work studied this problem using the boundary element method (*28*), quantization via the Selberg trace formula (*29*), time-dependent methods (*30*), and an algorithm based on the method of particular solutions (*31*). In accordance with our previous expectations, the spectrum {*E_n_*(0)} of −Δ with strictly automorphic boundary conditions is found to be real and discrete. For the Bolza surface of interest to us, the lowest eigenvalue is *E*_0_(0) = 0, corresponding to a constant eigenfunction over 𝒟, and the next three eigenvalues are given approximately by *E*_1_(0) ≈ 3.839, *E*_2_(0) ≈ 5.354, and *E*_3_(0) ≈ 14.726 (*31*).

**Fig. 2.**
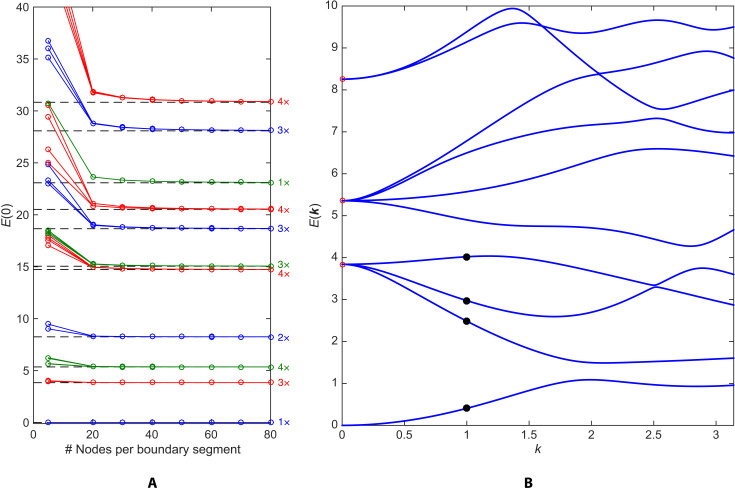
Hyperbolic bandstructure of the Bolza lattice in the empty-lattice approximation. (**A**) *k* = 0 eigenenergies computed using the finite element method (colored plots) versus eigenvalues of the Laplacian on the Bolza surface taken from (*31*) (dashed lines); only the lowest 11 distinct eigenvalues are shown [degeneracies from Strohmaier and Uski (*31*) are shown on the right]. The total number of mesh nodes grows approximately quadratically with the number of boundary nodes (see the Supplementary Materials). (**B**) Hyperbolic bandstructure plotted along a generic direction in the hyperbolic Brillouin zone: ***k*** = (*k*_1_, *k*_2_, *k*_3_, *k*_4_) = (0.8,0.3,1.2,1.7)*k*. Red circles: Eigenvalues of the Laplacian on the Bolza surface taken from Strohmaier and Uski (*31*). Black dots: Eigenstates whose probability densities are plotted in Fig. 3 (E to H).

Here, we study the general case ***k*** ≠ 0 for Σ_2_ using the finite element method. We use a freely available software package, FreeFEM++ (*32*), which was used successfully to study the spectrum of the Bolza surface with strictly automorphic boundary conditions (*33*). Our implementation of the twisted boundary conditions is discussed in the Supplementary Materials. As a check on our calculations, we first compute the spectrum {*E_n_*(0)}, i.e., with strictly automorphic boundary conditions (colored plots in Fig. 2A). With increased refinement of the finite element mesh, the ***k*** = 0 spectrum gradually converges to previously obtained results (*31*). In particular, the degeneracies found in previous studies (*31*, *33*) are correctly reproduced with a sufficiently fine mesh. Such degeneracies are a consequence of the large symmetry group (automorphism group) of the Bolza surface (*33*), which, as will be seen later, can be thought of as the hyperbolic analog of a point group. We use a mesh with 70 nodes per boundary segment in all remaining plots, which achieves satisfactory accuracy at reasonable computational cost. Because the spectrum is unbounded, we only compute a small number of low-lying eigenvalues using standard numerical linear algebra techniques.

A well-known result in conventional band theory is that, ignoring spin degrees of freedom, degeneracies at high-symmetry points fully split as one moves away from such points along a generic direction in reciprocal space (*2*). An example of a high-symmetry point is the origin ***k*** = 0 of the Brillouin zone—equivalently, the group identity in Jac(Σ_2_). To ascertain whether this behavior holds in the hyperbolic case, we compute the hyperbolic bandstructure for ***k*** ≠ 0 along a generic direction in the hyperbolic Brillouin zone (Fig. 2B). The lowest eigenvalue *E*_0_(0) = 0 is nondegenerate at ***k*** = 0 and thus does not split. As in the Euclidean case, the energy *E*_0_(***k***) of the lowest band increases with the magnitude of ***k*** at small ***k***, in accordance with the intuitive expectation that (kinetic) energy increases with crystal momentum in the long-wavelength limit. The next three eigenvalues *E*_1_(0), *E*_2_(0), and *E*_3_(0) are three-, four-, and twofold degenerate, respectively (*31*, *33*), but this degeneracy is completely lifted as ***k*** moves away from zero, as in conventional band theory. We also observe linear crossings between some of the bands emanating from *E*_2_(0) and *E*_3_(0). According to the von Neumann–Wigner theorem (*34*), only codimension-3 level crossings are perturbatively stable in the absence of symmetries other than translational. Thus, by contrast with 2D (or 3D) Euclidean lattices, we expect generically stable nodal-line crossings (*35*) in the hyperbolic bandstructures of {8,8} tessellations, and, for general {4*g*, 4*g*} tessellations, stable crossings forming (2*g* − 3)–dimensional submanifolds of Jac(Σ*_g_*).

In algebraic geometry, the points of degeneracy are known as ramification points, while the splitting off of eigensheets is known as branching. From this point of view, the total energy manifold *E_n_*(***k***), for all *n* and for all ***k***, is a branched cover of Jac(Σ_2_), although not one of finite type, as there are countably- but not finitely-many levels *n*. Finite-type branched covers arising from eigenvalues of finite-rank linear operators, known as spectral covers, are studied frequently in algebraic geometry, especially in connection with gauge theories, integrable systems, and high-energy physics, e.g., (*36*).

**Fig. 3.**
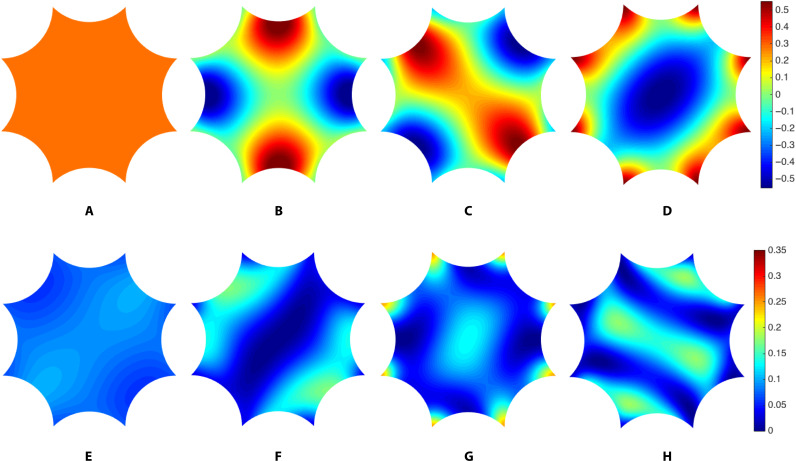
Hyperbolic Bloch eigenstates in the empty-lattice approximation. Wave function ψ*_k_*(*z*) for the (**A**) ground state and (**B** to **D**) degenerate first excited states at ***k*** = 0. Modulus squared ∣ψ_***k***_(*z*)∣^2^ for the (**E**) ground and (**F** to **H**) first three excited states corresponding to the black dots in Fig. 2B, i.e., at ***k*** = (0.8,0.3,1.2,1.7), in order of increasing eigenenergy.

Our finite element calculation also gives us access to the detailed spatial profile of the hyperbolic Bloch wave functions ψ_*n****k***_(*z*). Because these wave functions obey the automorphic Bloch condition (2) by construction, it is sufficient to plot them for *z* in the central hyperbolic octagon 𝒟 (Fig. 3). At ***k*** = 0, the wave functions are purely real. The ground state (Fig. 3A) is nodeless and perfectly uniform, while the excited states (Fig. 3, B to D) acquire nodes. For ***k*** ≠ 0, the wave functions are generally complex, as in the Euclidean case. The probability densities for ground and excited states (Fig. 3, E to H) are modulated by the ***k*** vector with respect to their ***k*** = 0 counterparts. [Note, however, that the three excited states in Fig. 3 (B to D) are degenerate and only represent one possible basis of the degenerate subspace, which is split at ***k*** ≠ 0; thus, one cannot directly match Fig. 3 (B to D) and Fig. 3 (F to H).]

### A particle in an automorphic potential

We now consider turning on a nonzero automorphic potential *V*. Such a potential can be constructed by summing over all Γ-translates of a localized potential *U*(*z*)

$$V(z)=\sum_{\gamma \in \Gamma }U(\gamma (z))$$ (5)

which is a kind of generalized theta series (*13*). To ensure that this series converges everywhere, we choose *U*(*z*) with compact support in 𝒟, for instance, a circular well of radius *R* and depth *V*_0_. Because the full Hamiltonian *H* = − Δ + *V* is invariant under Γ-translations, it is sufficient to solve the Schrödinger equation (Eq. 3) with the automorphic Bloch boundary conditions (Eq. 2) on 𝒟.

**Fig. 4.**
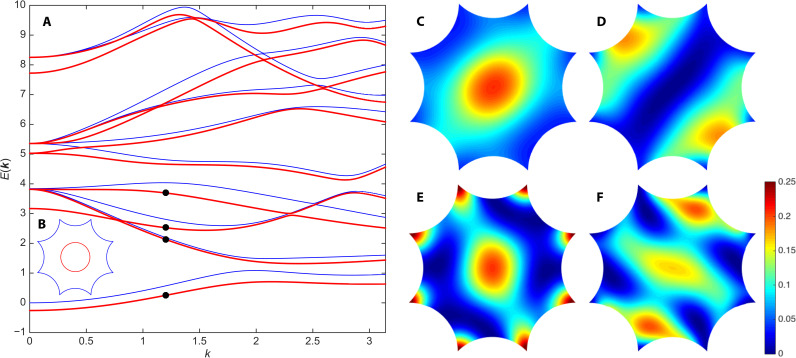
Hyperbolic Bloch problem with nontrivial automorphic potential of Eq. 5. (**A**) Bandstructure with (red) and without (blue) automorphic potential, along the same direction in the hyperbolic Brillouin zone as in Fig. 2. (**B**) Circularly symmetric potential *U*(*z*) in the octagonal unit cell, with *R* = 0.3 and *V*_0_ = 2. (**C** to **F**) Modulus squared ∣ψ_***k***_(*z*)∣^2^ of the eigenstates corresponding to the black dots in (A), i.e., at ***k*** = (0.8,0.3,1.2,1.7)*k*, with *k* = 1.2, in increasing order of eigenenergy.

In Fig. 4A, we plot the hyperbolic bandstructure for the potential (Eq. 5) with *R* = 0.3 and *V*_0_ = 2, illustrated schematically in the inset (Fig. 4B). Focusing first on the ***k*** = 0 eigenenergies, we find that the ground-state energy is lowered from *E*_0_(0) = 0 to a negative value, as expected for an attractive potential. We observe a (partial) lifting of the ***k*** = 0 degeneracies: the threefold degeneracy of *E*_1_(0) is split as 2 ⊕ 1; the fourfold degeneracy of *E*_2_(0), as 2 ⊕ 2; and the twofold degeneracy of *E*_3_(0) is lifted. For both the first and second excited spectral manifolds, we find that the energy of one of the doublets is virtually unchanged from the original unperturbed eigenvalue. For the first excited manifold, this can be understood from the fact that, for two of the three unperturbed eigenstates (Fig.3, B and C), most of the probability density is concentrated near the boundary segments, with very little near the center of the octagon. From the perspective of degenerate perturbation theory, the average of the potential *U*(*z*) over the appropriate linear combinations would yield a small correction to the eigenenergies. By contrast, the third unperturbed eigenfunction (Fig. 3D) has modulus squared peaked near the center of the octagon and also at its corners: It registers the potential more, and the correction to its eigenenergy is correspondingly greater.

In Fig. 4 (C to F), we plot the modulus squared of the hyperbolic Bloch wave functions corresponding to the (nondegenerate) levels indicated by black dots in Fig. 4A. For the lowest band (Fig. 4C), due to the attractive potential, the probability density is much more concentrated near the center of the unit cell, as compared to the empty-lattice approximation (e.g., Fig. 3E), although the value of ***k*** is not exactly the same. The eigenfunctions for the next three bands (Fig. 4, D to F) are also distorted with respect to their empty-lattice counterparts (Fig. 3, F to H). The observation that the probability density in Fig. 4E is peaked near the center and at the corners of the octagonal unit cell—combined with the fact that, at ***k*** = 0, this same hyperbolic Bloch state belongs to the singlet in the splitting 3 → 2 ⊕ 1 discussed above for the *E*_1_(0) spectral manifold—confirms our earlier speculation concerning the qualitative reason for this splitting.

### Hyperbolic point-group symmetries

We have so far only discussed the hyperbolic analog of lattice translations, namely, elements of a co-compact, strictly hyperbolic Fuchsian group Γ ⊂ *PSU*(1,1). Like Euclidean translations, these elements act on the hyperbolic plane without fixed points and are essentially 2D Lorentz boosts (*16*). In addition, akin to Euclidean lattices, hyperbolic lattices admit the analog of point-group symmetries, which are discrete symmetries that leave at least one point of the lattice fixed. A complete hyperbolic band theory must also include a discussion of these, with particular attention paid to how such point-group symmetries manifest in ***k***-space.

For a 2D Euclidean lattice, the point group *G* is a finite subgroup of the orthogonal group *O*(2), which includes *SO*(2) rotations but also orientation-reversing transformations, that is, reflections. Point-group symmetries imply that if ψ_***k***_(***r***) is a Bloch eigenstate for such a lattice with energy *E*(***k***), the transformed state $\psi_{k}^{h}(r)\equiv \psi_{k}(hr)$, with *h* ∈ *G*, is also an eigenstate with the same energy. By elementary properties of Fourier transforms, this transformed state is a Bloch state with wave vector ***k****^h^* ≡ *h****k***, which implies that the bandstructure must obey *E*(*h****k***) = *E*(***k***).

In the absence of an Abelian translation group, Fourier transforms cannot be directly used to generalize these ideas to hyperbolic lattices. Furthermore, because for a {4*g*, 4*g*} hyperbolic lattice ***k***-space is 2*g*-dimensional, the very question of how nontranslational discrete symmetries in 2D hyperbolic space act in a higher-dimensional ***k***-space is a deep conceptual one. That said, given that the Abel-Jacobi map provides an algebraic replacement for the Fourier transform, this duality provides a potentially lucrative route for exploring the effect of point-group symmetries. As the group acts on Σ*_g_*, it acts on the symmetric product of Σ*_g_* with itself and, hence, on Jac(Σ*_g_*) via Abel-Jacobi. As the action moves the points *p*_1_, …, *p_g_* in Σ*_g_*, it moves the end points of the paths of integration in the definition (Eq. 4) of map *a*, which is the induced action on Jac(Σ*_g_*). We recall that there is a high-symmetry region within the Jacobian—in this region, the action may have more fixed points. We aim to use this point of view in further work.

For the specific case of the Bolza curve, we are able to examine the point-group action directly. Via concrete calculations for the Bolza lattice, we argue in the Supplementary Materials that the proper generalization of point group for {4*g*, 4*g*} hyperbolic lattices is the finite group *G* ≅ Aut(Σ*_g_*) of automorphisms (i.e., self-maps) of the genus-*g* Riemann surface (*18*) associated with the compactified 4*g*-gonal unit cell. For the Bolza surface, it is a non-Abelian group of order 96 generated by four Möbius transformations (*33*): an eightfold rotation (*R*) around the center of the octagon and a threefold rotation-like operation (*U*), both orientation preserving, and two reflection-like operations (*S* and *T*), both orientation reversing. Furthermore, as in the Euclidean case, we find that this hyperbolic point group acts linearly on hyperbolic ***k***-space: ***k****^h^* = *M*(*h*)***k***, *h* ∈ *G*, where the 4 × 4 matrices *M*(*h*), *h* ∈ *G*, form an *SL*(4, ℤ) representation of *G*. Explicit representation matrices for the generators *h* = *R*, *S*, *T*, *U*, from which the representation matrix of any element of *G* can be constructed by matrix multiplication, are given in the Supplementary Materials. For a {4*g*, 4*g*} hyperbolic lattice, we conjecture that ***k*** transforms similarly, with *M* a representation of *G* valued in *SL*(2*g*, ℤ). By contrast with the Euclidean case, however, the matrices *M*(*h*) are, in general, not orthogonal and thus do not simply correspond to the action of a Euclidean point group in 2*g* dimensions.

**Fig. 5.**
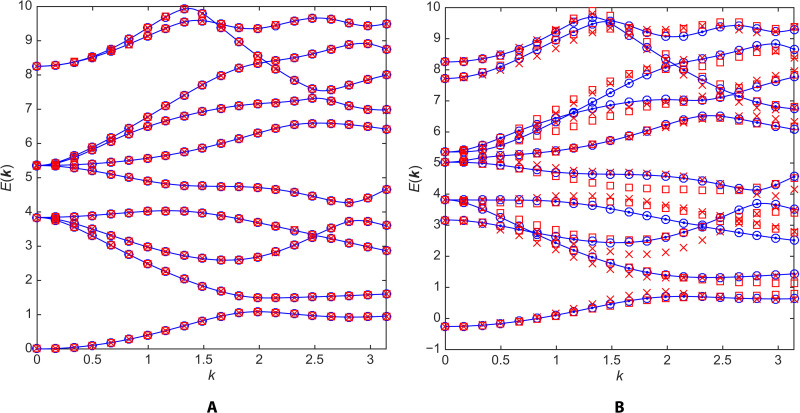
Point-group symmetries in hyperbolic *k*-space. (**A**) Empty-lattice approximation and (**B**) automorphic potential of Fig. 4. Blue lines: Hyperbolic bandstructure *E_n_*(***k***) along the direction of Figs. 2 and 4. Blue dots: *E_n_*(***k****^R^*). Blue circles: *E_n_*(***k****^S^*). Red crosses: *E_n_*(***k****^T^*). Red squares: *E_n_*(***k****^U^*).

In Fig. 5A, we verify numerically that the hyperbolic bandstructure in the empty-lattice approximation is invariant under the full hyperbolic point group *G* of the Bolza lattice, meaning that *E_n_*(***k****^h^*) = *E_n_*(***k***) for all *h* ∈ *G*. We choose ***k*** along the generic direction already considered in Fig. 2 and plot both *E_n_*(***k***) (blue lines) and *E_n_*(***k****^h^*) (colored symbols), where ***k****^h^* is the direction related to ***k*** by point-group symmetry *h*. We verify that the bandstructure is left unchanged under the action of all four generators *h* = *R*, *S*, *T*, *U* of *G*, thus establishing invariance under the full point group.

Figure 5A is to be contrasted with Fig. 5B, which illustrates that the automorphic potential chosen in Eq. 5 breaks at least some of the hyperbolic point-group symmetries of the Bolza lattice. The *R* operation is a π/4 rotation about the origin, and the *S* operation is a reflection across the *x* axis followed by a π/4 rotation. Although formally defined as Möbius transformations, they reduce to simple Euclidean isometries that are obvious symmetries of a circular potential well. As a result, the bandstructure is left unchanged under ***k*** → ***k****^h^* with *h* = *R*, *S*. By contrast, the *T* and *U* operations are genuine non-Euclidean isometries involving boosts (see the Supplementary Materials) that do not leave the potential invariant. Correspondingly, the bandstructure does not exhibit invariance under ***k*** → ***k****^h^*, with *h* = *T*, *U*.

### The tight-binding limit

In conventional band theory, the tight-binding method is a commonly used approximation scheme to analyze the Schrödinger equation in the limit of deep periodic potentials (*2*). While inexact, it provides a conceptually important, and often sufficiently accurate, framework to study the Bloch problem in this limit. The tight-binding method starts from the discrete spectrum and localized eigenstates of isolated potential wells and builds on the idea that propagation throughout the crystal proceeds via weak quantum tunneling between those localized states. Our hyperbolic band theory described so far is based on the full Schrödinger equation and applies to arbitrary {4*g*, 4*g*} automorphic potentials, including deep ones. However, to further develop our generalization of band theory and in light of the experiments of Kollár *et al*. (*7*), which are most simply modeled using the tight-binding method, it is natural to ask whether an explicit tight-binding formulation of hyperbolic band theory can be devised. In the Supplementary Materials, we show that this is indeed possible. In the limit of a deep localized potential *U*(*z*), approximate eigenstates that obey the automorphic Bloch condition (2) can be constructed as linear combinations of eigenstates of the “atomic” problem −Δ + *U* and their Γ-translates, in the spirit of the linear combination of atomic orbitals familiar in solid-state physics (*2*). The coefficients of this expansion are eigenvectors of a finite-dimensional ***k***-dependent matrix eigenvalue problem, the dimension of which is equal to the number of atomic eigenstates kept in the expansion, and the eigenvalues produce an approximate hyperbolic bandstructure. A hyperbolic analog of Wannier functions can likewise be constructed (see the Supplementary Materials).

Our work opens up several exciting avenues of research. While we have shown how to construct a continuous family of Bloch eigenstates for a large class of Hamiltonians with the symmetry of a hyperbolic tessellation, we have not provided a hyperbolic equivalent of Bloch’s theorem—that is, a statement that all eigenstates of the Hamiltonian are hyperbolic Bloch eigenstates. What precise fraction of the full spectrum is captured by the hyperbolic Bloch family of eigenstates and the nature of those eigenstates that may not be of hyperbolic Bloch form are thus important questions for future research. One obvious line of attack is to attempt to match our predictions with those obtained from numerical diagonalization on {4*g*, 4*g*} lattices, keeping in mind possible subtle issues related to the implementation of automorphic boundary conditions in finite lattices, especially given the different relative importance of bulk versus boundary in Euclidean versus hyperbolic geometries. It may also be possible to approach those spectral questions using number-theoretic tools such as the Selberg trace formula and associated zeta function (*21*, *37*). Even within the Bloch condition, the role of factors of automorphy of nonzero weight is an intriguing question in the hyperbolic setting.

In further pursuing the connections to algebraic geometry and number theory, we note that higher-dimensional versions of our construction may be produced now for K3 surfaces and Calabi-Yau manifolds [e.g., (*38*)], which generalize elliptic curves, and for Shimura varieties [e.g., (*39*)], which generalize modular curves. Working over Calabi-Yau manifolds is especially tantalizing as a potential pathway for novel connections between high-energy physics and condensed matter, which may offer new tools to the latter from string theory and mirror symmetry [e.g., (*40*)]. In three spatial dimensions specifically, we also anticipate connections with the work of Thurston (*41*), whereby hyperbolic bandstructures may arise in connection with 3D hyperbolic tessellations, their (Kleinian) groups of discrete translations, and the geometry and topology of compact three manifolds produced by the quotienting of 3D hyperbolic space by Kleinian translations. Last, on the experimental side, we advocate the fabrication and characterization of {4*g*, 4*g*} lattices using circuit quantum electrodynamics (*7*), photonic (*12*), or other metamaterial platforms.

Our construction carries with it a realization that our topological understanding of condensed matter is a small corner of a theory that is perhaps, by and large, algebro-geometric in nature. Our construction anticipates the emergence of algebro-geometric invariants alongside topological ones, such as Donaldson-Thomas invariants (*42*) of higher-dimensional complex varieties.

## Supplementary Materials

### This PDF file includes:

Sections S1 to S6 Figs. S1 and S2 References
