# Supplementary material for: Hyperbolic band theory
Source: Sci Adv. 2021 Sep 3;7(36):eabe9170. doi: 10.1126/sciadv.abe9170 (PMC8442860; doi:10.1126/sciadv.abe9170)
Supplement: Supplementary file 1 — Sections S1 to S6 Figs. S1 and S2 References [file sciadv.abe9170_sm.pdf]

Science Advances
AAAS

Supplementary Materials for

Hyperbolic band theory

Joseph Maciejko* and Steven Rayan*

*Corresponding author. Email: maciejko@ualberta.ca (J.M.); rayan@math.usask.ca (S.R.)

Published 3 September 2021, *Sci. Adv.* **7**, eabe9170 (2021)
DOI: 10.1126/sciadv.abe9170

This PDF file includes:

Sections S1 to S6
Figs. S1 and S2
References

S1. GEOMETRY OF THE BOLZA LATTICE

In this Section, we detail the geometry of the regular $\{8,8\}$ hyperbolic tessellation illustrated in Fig. 1(c) of the main text. This tessellation is composed of repeated regular hyperbolic octagons, eight of which meet at each vertex of the lattice. To fully describe the tessellation, it is sufficient to give the geometry of a reference unit cell $\mathcal{D}$, here chosen to be the octagon centered at the origin $z = 0$ of the Poincaré disk, and the $PSU(1,1)$ matrix representation of the noncommutative Fuchsian group Γ that translates $\mathcal{D}$ to cover the entire Poincaré disk.

To describe $\mathcal{D}$, we give its boundary segments. These are (circular) geodesic arcs normal to the boundary at infinity $|z| = 1$, and can be parametrized as follows:

$$C_j = \left\{ z = e^{i(j-1)\frac{\pi}{4}}(c + re^{i\theta}), \frac{3\pi}{4} < \theta < \frac{5\pi}{4} \right\}, \qquad j = 1, \ldots, 8, \tag{S1}$$

where

$$c = \sqrt{\frac{3+2\sqrt{2}}{2+2\sqrt{2}}}, \qquad r = \frac{1}{\sqrt{2+2\sqrt{2}}}. \tag{S2}$$

The vertices of $\mathcal{D}$ are given by

$$p_j = 2^{-1/4} e^{i(2j-1)\frac{\pi}{8}}, \qquad j = 1, \ldots, 8. \tag{S3}$$

An explicit $PSU(1,1)$ matrix representation of the four generators γ_1, γ_2, γ_3, γ_4 which generate Γ is given in Ref. [30]:

$$\gamma_j = \begin{pmatrix} 1+\sqrt{2} & (2+\sqrt{2})\lambda e^{i(j-1)\pi/4} \\ (2+\sqrt{2})\lambda e^{-i(j-1)\pi/4} & 1+\sqrt{2} \end{pmatrix}, \qquad j = 1, \ldots, 4, \tag{S4}$$

where $\lambda = \sqrt{\sqrt{2}-1}$. One can check by explicit computation that the action of those generators identifies the boundary segments pairwise and in an orientation-preserving manner, as depicted in Fig. 1(c) of the main text:

$$\gamma_1(C_5) = C_1, \qquad \gamma_2(C_6) = C_2, \qquad \gamma_3(C_7) = C_3, \qquad \gamma_4(C_8) = C_4. \tag{S5}$$

The inverse Möbius transformations γ_j^{-1} correspond simply to the matrix inverse of Eq. (S4). The generators of Γ obey the relation

$$\gamma_1\gamma_2^{-1}\gamma_3\gamma_4^{-1}\gamma_1^{-1}\gamma_2\gamma_3^{-1}\gamma_4 = \mathbb{I}, \tag{S6}$$

where $\mathbb{I}$ again denotes the identity. As shown in Sec. S2, Eq. (S6) is precisely the presentation of the fundamental group $\pi_1(\Sigma_2)$ of a smooth, compact genus-2 surface, illustrated schematically in Fig. 1(d) of the main text, that is obtained by gluing together the opposite sides of the hyperbolic octagon $\mathcal{D}$. This establishes the group isomorphism $\pi_1(\Sigma_2) \cong \Gamma$. We emphasize that while all genus-2 topological surfaces share this fundamental group, the symbol Σ_2 refers not to *any* genus-2 surface but specifically to the quotient $\mathbb{H}/\Gamma$ for the Γ generated above. This quotient is precisely the side identification illustrated in Fig. 1(c) of the main text. To use our earlier language, Σ_2 is a particular Riemann surface structure defined upon a topological genus-2 surface. This Riemann surface is known traditionally as the *Bolza surface* [43]. Accordingly, we shall refer to the regular $\{8,8\}$ tessellation as the *Bolza lattice*. The Bolza surface is but one Riemann surface in an entire *moduli space* of distinct Riemann surfaces of genus 2 [44]. In general, there are $3g-3$ complex degrees of freedom to vary the Riemann surface structure whenever $g \geqslant 2$—this is the dimension of the moduli space—while the underlying topological class has no freedom. Of the points in the moduli space, the Bolza surface is the Riemann surface of genus 2 with the largest possible automorphism group, reflecting the high degree of symmetry in the original lattice and its edge identifications. (We use automorphisms of Riemann surfaces to generalize the notion of point-group symmetries to hyperbolic lattices in Sec. S4.)

S2. FUNDAMENTAL GROUP OF THE BOLZA SURFACE

The Bolza surface Σ_2 is a Riemann surface of genus 2 [43] obtained by identifying the opposite sides of the hyperbolic octagon in Fig. 1(c) of the main text. Under this identification, the eight vertices $p_1,\ldots,p_8$ of the octagon are mapped to a single point p_0 which we can take as the base point for loops [14]. Under the identification, each of the eight geodesic boundary segments $C_1,\ldots,C_8$ starts and ends at p_0 and is thus a closed loop; thus the C_j, $j=1,\ldots,8$ are elements of the fundamental group $\pi_1(\Sigma_2,p_0)$ based at p_0. Consider a closed path C that starts at $p_1 \sim p_0$ and goes around the boundary of the octagon counterclockwise. Denoting by $C_1,\ldots,C_8$ the oriented paths with orientations indicated by the arrows in Fig. 1(c) of the main text, and by $C_1^{-1},\ldots,C_8^{-1}$ the same paths traversed in reverse, C is given by

$$C = C_1C_2^{-1}C_3C_4^{-1}C_5^{-1}C_6C_7^{-1}C_8. \tag{S7}$$

Now, C can be continuously deformed to a point inside the octagon, thus it is homotopic to the trivial path: $C = 1$. This remains true after identification. After identification, however, C_j and C_{j+4}, $j = 1, \ldots, 4$ are identified in an orientation-preserving manner. Therefore, the unique relation satisfied by the distinct generators C_1, C_2, C_3, C_4 of $\pi(\Sigma_2, p_0)$ is:

$$C_1 C_2^{-1} C_3 C_4^{-1} C_1^{-1} C_2 C_3^{-1} C_4 = 1. \tag{S8}$$

Since fundamental groups with different base points are isomorphic, we can simply write:

$$\pi_1(\Sigma_2) = \{C_1, C_2, C_3, C_4 : C_1 C_2^{-1} C_3 C_4^{-1} C_1^{-1} C_2 C_3^{-1} C_4 = 1\}. \tag{S9}$$

Isomorphic groups may have different, but equivalent, presentations. For example, one can give a different presentation for (S9) as follows [30]. Define new generators as $a_1 = C_3$, $b_1 = C_4^{-1}$, $a_2 = C_1 C_2^{-1}$, and $b_2 = C_3 C_4^{-1} C_1^{-1}$. Then using (S8), one finds that the relation satisfied by these new generators is that quoted in the main text, i.e.,

$$\pi_1(\Sigma_g) \cong \{a_1, b_1, \ldots, a_g, b_g : a_1 b_1 a_1^{-1} b_1^{-1} \cdots a_g b_g a_g^{-1} b_g^{-1} = 1\}, \tag{S10}$$

with $g = 2$. Correspondingly, the original generators can be obtained from the new ones by $C_1 = b_2^{-1} a_1 b_1$, $C_2 = a_2^{-1} b_2^{-1} a_1 b_1$, $C_3 = a_1$, and $C_4 = b_1^{-1}$. Since products of homotopy classes correspond to composition of loops, different choices of generators correspond to different choices of closed loops on the Riemann surface. In the section "The Bolza lattice" in the main text, our choice of representation $\chi : \pi_1(\Sigma_2) \to U(1)$,

$$\chi(\gamma_j) = \chi(\gamma_j^{-1})^* = e^{ik_j}, \qquad j = 1, \ldots, 4, \tag{S11}$$

associates k_1, k_2, k_3, k_4 with the Aharonov-Bohm phase acquired upon traversing C_1, C_2, C_3, C_4, but the choice

$$\chi(a_i) = \chi(a_i^{-1})^* = e^{ik_a^{(i)}}, \qquad \chi(b_i) = \chi(b_i^{-1})^* = e^{ik_b^{(i)}}, \qquad i = 1, \ldots, g, \tag{S12}$$

with a_1, b_1, a_2, b_2 defined above, is equally valid. It can simply be thought of as the choice of a different basis for the hyperbolic reciprocal lattice, i.e., the $2g$-dimensional lattice Λ such that the quotient $\mathbb{R}^{2g} \cong \mathbb{C}^g$ by it gives $\mathrm{Jac}(\Sigma_g) \cong T^{2g}$. We also note that since the representation χ is abelian, it automatically satisfies the group relation in $\pi_1(\Sigma_2)$.

S3. TWISTED AUTOMORPHIC BOUNDARY CONDITIONS IN THE FINITE ELEMENT METHOD

The FreeFEM++ package [32] allows for Dirichlet, Neumann, or strictly periodic/automorphic boundary conditions, but not directly for the twisted automorphic boundary conditions required for nonzero $\boldsymbol{k}$, i.e.,

$$\psi(C_j) = e^{ik_j} \psi(C_{j+4}), \qquad j = 1, \ldots, 4. \tag{S13}$$

To remedy this problem, we follow the approach of Refs. [45–47], in which the stiffness and overlap matrices of the weak (variational) formulation of the hyperbolic Schrödinger equation [Eq. (3) in the main text] are first computed using FreeFEM++ with unconstrained boundary conditions, and the number of physical degrees of freedom is subsequently reduced using simple matrix operations before proceeding to numerical diagonalization. The generalized Bloch phases are easily introduced at this second stage, as we now explain.

Consider two functions ϕ, ψ obeying the twisted automorphic condition [Eq. (2) in the main text]. The weak form of the Schrödinger equation is obtained by multiplying this equation by ϕ^* and integrating over the domain $\mathcal{D}$,

$$\int_{\mathcal{D}} d^2r\sqrt{g}\,(-\phi^*\Delta\psi + \phi^* V\psi) = E\int_{\mathcal{D}} d^2r\sqrt{g}\phi^*\psi, \tag{S14}$$

where $\sqrt{g} = 4/(1-|z|^2)^2$ is the square root of the determinant of the Poincaré metric tensor $g_{\mu\nu} = 4\delta_{\mu\nu}/(1-|z|^2)^2$, and $d^2r = dx\,dy$ is the usual Euclidean integration measure. Using Green's theorem and the automorphic Bloch condition, one can show that Eq. (S14) becomes [21]

$$\int_{\mathcal{D}} d^2r\sqrt{g}\,(g^{\mu\nu}\partial_\mu\phi^*\partial_\nu\psi + \phi^* V\psi) = E\int_{\mathcal{D}} d^2r\sqrt{g}\phi^*\psi. \tag{S15}$$

Using the inverse metric tensor $g^{\mu\nu} = \frac{1}{4}\delta^{\mu\nu}(1-|z|^2)^2$, we obtain

$$\int_{\mathcal{D}} d^2r\left(\partial_x\phi^*\partial_x\psi + \partial_y\phi^*\partial_y\psi + \frac{4\phi^* V\psi}{(1-|z|^2)^2}\right) = E\int_{\mathcal{D}} d^2r\frac{4\phi^*\psi}{(1-|z|^2)^2}. \tag{S16}$$

In the finite element method, one triangulates the region $\mathcal{D}$ (e.g., Fig. S1) and expands the solution ψ on a basis of functions u_i, $i = 1, \dots, M$ with compact support on each finite element (i.e., triangle) in the triangulation. While M is formally infinite, in practice, we truncate $\{u_i\}$ to the set of linear Lagrangian shape functions (P1 elements in the notation of Ref. [32]). M then equals the finite number of vertices in the triangulation, and the piecewise-linear basis function u_i equals one on vertex i and vanishes on all other vertices. While simple, the choice of P1 elements allows us to achieve satisfactory accuracy with a sufficiently fine triangulation. Expanding $\psi = \sum_{j=1}^{M}\psi_j u_j$ and taking $\phi = u_i$, Eq. (S16) becomes

$$\sum_{j=1}^{M} A_{ij}\psi_j = E\sum_{j=1}^{M} B_{ij}\psi_j, \qquad i = 1, \dots, M, \tag{S17}$$

where the $M \times M$ Hermitian matrices A (stiffness matrix) and B (overlap matrix) are

$$A_{ij} = \int_{\mathcal{D}} d^2r\left(\partial_x u_i^*\partial_x u_j + \partial_y u_i^*\partial_y u_j + \frac{4u_i^* V u_j}{(1-|z|^2)^2}\right), \qquad B_{ij} = \int_{\mathcal{D}} d^2r\frac{4u_i^* u_j}{(1-|z|^2)^2}. \tag{S18}$$

The solution of the Schrödinger equation in continuous space is thus approximated by the solution of a finite-dimensional generalized eigenvalue problem, Eq. (S17).

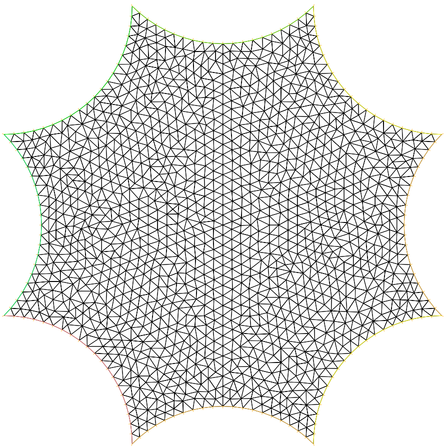

FIG. S1. A finite element triangulation of the hyperbolic octagon $\mathcal{D}$ with 20 nodes per boundary segment.

We now explain how to impose the boundary conditions (S13). Since i, j are vertex indices, the solution vector $\boldsymbol{\psi} = (\psi_1, \ldots, \psi_M)$ can be written in block form as

$$\boldsymbol{\psi} = (\psi_{p_1}, \ldots, \psi_{p_8}; \boldsymbol{\psi}_{C_1}, \ldots, \boldsymbol{\psi}_{C_8}; \boldsymbol{\psi}_{\text{bulk}}) , \tag{S19}$$

in the notation of Fig. 1(c), where $p_1, \ldots, p_8$ denote vertices at the eight corners of the hyperbolic octagon; $\boldsymbol{\psi}_{C_j}$, $j = 1, \ldots, 8$, is a vector containing the solution on the set of vertices belonging to the boundary segment C_j of the octagon, excluding corners; and $\boldsymbol{\psi}_{\text{bulk}}$ is a vector containing the solution on the interior vertices. The boundary conditions, which identify boundary segments (including corners) pairwise, imply that only $N < M$ degrees of freedom are in fact independent. There thus exists an $M \times N$ matrix U such that $\boldsymbol{\psi} = U\tilde{\boldsymbol{\psi}}$, where $\tilde{\boldsymbol{\psi}}$ is an N-dimensional vector containing only the independent degrees of freedom. Substituting this equation inside the M-dimensional generalized eigenvalue problem $A\boldsymbol{\psi} = EB\boldsymbol{\psi}$ in (S17), and left-multiplying by $U^\dagger$, we obtain the reduced, N-dimensional generalized eigenvalue problem:

$$\tilde{A}\tilde{\boldsymbol{\psi}} = E\tilde{B}\tilde{\boldsymbol{\psi}}, \tag{S20}$$

where

$$\tilde{A} = U^\dagger A U, \qquad \tilde{B} = U^\dagger B U, \tag{S21}$$

are again Hermitian.

The $M \times N$ matrix U is constructed as follows. First, the bulk vertices are unaffected by the boundary conditions, thus $\boldsymbol{\psi}_{\text{bulk}}$ appears in full in $\tilde{\boldsymbol{\psi}}$. Next, out of the eight boundary vectors $\boldsymbol{\psi}_{C_1}, \ldots, \boldsymbol{\psi}_{C_8}$, only four are linearly independent due to the boundary conditions. Using Eq. (S13),

we can thus express all eight boundary vectors in terms of $\boldsymbol{\psi}_{C_5}, \ldots, \boldsymbol{\psi}_{C_8}$:

$$\begin{pmatrix} \boldsymbol{\psi}_{C_1} \\ \boldsymbol{\psi}_{C_2} \\ \boldsymbol{\psi}_{C_3} \\ \boldsymbol{\psi}_{C_4} \\ \boldsymbol{\psi}_{C_5} \\ \boldsymbol{\psi}_{C_6} \\ \boldsymbol{\psi}_{C_7} \\ \boldsymbol{\psi}_{C_8} \end{pmatrix} = \begin{pmatrix} e^{ik_1} & & & \\ & e^{ik_2} & & \\ & & e^{ik_3} & \\ & & & e^{ik_4} \\ 1 & & & \\ & 1 & & \\ & & 1 & \\ & & & 1 \end{pmatrix} \begin{pmatrix} \boldsymbol{\psi}_{C_5} \\ \boldsymbol{\psi}_{C_6} \\ \boldsymbol{\psi}_{C_7} \\ \boldsymbol{\psi}_{C_8} \end{pmatrix}, \tag{S22}$$

making also sure that the components of the respective vectors are ordered so as the preserve orientation under pairwise identification [see Fig. 1(c) in the main text]. Finally, all eight corner vertices $p_1, \ldots, p_8$ collapse under this identification to a single point, which can be chosen as p_5. Using the action of the Fuchsian group generators depicted in Fig. 1(c) of the main text, we can express the remaining vertices as:

$$\begin{aligned} &p_1 = \gamma_1\gamma_4\gamma_3^{-1}\gamma_2(p_5), \qquad p_2 = \gamma_2(p_5), \qquad p_3 = \gamma_4\gamma_1(p_5), \qquad p_4 = \gamma_4\gamma_3^{-1}\gamma_2(p_5), \\ &p_6 = \gamma_3^{-1}\gamma_4\gamma_1(p_5), \qquad p_7 = \gamma_3^{-1}\gamma_2(p_5), \qquad p_8 = \gamma_1(p_5). \end{aligned} \tag{S23}$$

Note that this choice of representation is not unique, but different representations can be shown to be equivalent using the relation (S6). Using the generalized Bloch factor (S11), we can thus write

$$\begin{pmatrix} \psi_{p_1} \\ \psi_{p_2} \\ \psi_{p_3} \\ \psi_{p_4} \\ \psi_{p_5} \\ \psi_{p_6} \\ \psi_{p_7} \\ \psi_{p_8} \end{pmatrix} = \begin{pmatrix} e^{i(k_1+k_2-k_3+k_4)} \\ e^{ik_2} \\ e^{i(k_1+k_4)} \\ e^{i(k_2-k_3+k_4)} \\ 1 \\ e^{i(k_1-k_3+k_4)} \\ e^{i(k_2-k_3)} \\ e^{ik_1} \end{pmatrix} \psi_{p_5}. \tag{S24}$$

Defining the reduced vector $\tilde{\boldsymbol{\psi}}$ as

$$\tilde{\boldsymbol{\psi}} = (\psi_{p_5}; \boldsymbol{\psi}_{C_5}, \ldots, \boldsymbol{\psi}_{C_8}; \boldsymbol{\psi}_{\text{bulk}}), \tag{S25}$$

and using Eqs. (S22) and (S24), as well as an identity matrix for $\boldsymbol{\psi}_{\text{bulk}}$, the matrix U can be easily constructed. Solving the generalized eigenvalue problem (S20) numerically for each $\boldsymbol{k}$ in the hyperbolic Brillouin zone, using standard linear algebra techniques, we obtain the hyperbolic bandstructure $\{E_n(\boldsymbol{k})\}$ and hyperbolic Bloch wavefunctions $\psi_{n\boldsymbol{k}}(x, y)$.

S4. POINT-GROUP SYMMETRIES OF THE BOLZA LATTICE

In ordinary crystallography, the group of all discrete symmetries of a Euclidean (periodic) lattice constitutes the space group $\mathcal{G}$. The translation group $\mathcal{T}$ is a normal subgroup of $\mathcal{G}$, that is, if $h \in \mathcal{G}$, then $h\mathcal{T}h^{-1} = \mathcal{T}$. The point group $\mathcal{P}$ is the factor group $\mathcal{P} \cong \mathcal{G}/\mathcal{T}$, i.e., space group operations with translations factored out [48]. Similarly, the group $G = \mathrm{Aut}(X)$ of automorphisms of a compact Riemann surface $X \cong \mathbb{H}/\Gamma$ with Γ a co-compact, strictly hyperbolic Fuchsian group is isomorphic to the factor group [18]

$$G \cong N(\Gamma)/\Gamma, \tag{S26}$$

where $N(\Gamma)$ is the normalizer of Γ in $PSL(2,\mathbb{R})$:

$$N(\Gamma) = \{h \in PSL(2,\mathbb{R}) : h\Gamma h^{-1} = \Gamma\}. \tag{S27}$$

By analogy with Euclidean crystallography, it is natural to intepret $N(\Gamma)$ as a hyperbolic space group, its normal subgroup Γ as a (nonabelian) translation group, and the factor group (S26) as a point group. Note that in Riemann surface theory [18], one is typically interested in orientation-preserving automorphisms, thus $N(\Gamma)$ is defined as the normalizer of Γ in $\text{Möb}^+ \cong PSL(2,\mathbb{R})$, the group of orientation-preserving Möbius transformations. Here we consider all automorphisms [33], both Möb^+ and orientation-reversing ones (Möb^-), and $N(\Gamma)$ is more properly defined as the normalizer of Γ in the general Möbius group Möb.

In this Section, we describe the group $G \cong \mathrm{Aut}(\Sigma_2)$ of automorphisms of the Bolza surface (Sec. S4 1), interpreting it as a point group, and construct its linear action

$$\boldsymbol{k} \to \boldsymbol{k}^h = M(h)\boldsymbol{k}, \qquad h \in G, \tag{S28}$$

on the Jacobian, i.e., hyperbolic $\boldsymbol{k}$-space (Sec. S4 2).

1. Automorphisms of the Bolza surface

The automorphism group G of the Bolza surface is a finite nonabelian group with 96 elements, of the form [33]

$$R^i S^j T^k U^l, \qquad i = 0,\dots,7, \qquad j,k = 0,1, \qquad l = 0,1,2, \tag{S29}$$

where R, S, T, U are four generators. R and U are orientation-preserving hyperbolic isometries, i.e., Möbius transformations of the form

$$z \to \gamma(z) = \frac{\alpha z + \beta}{\beta^* z + \alpha^*}. \tag{S30}$$

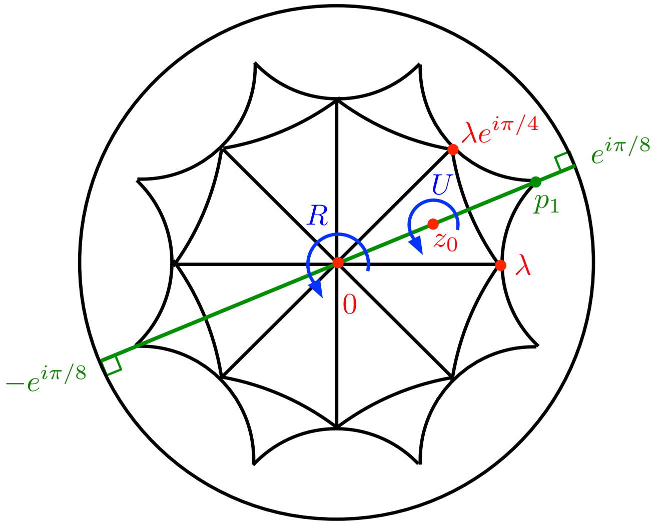

FIG. S2. Automorphisms of the Bolza surface (adapted from Ref. [33] with permission of the author).

Working in the Poincaré disk, the group Möb$^+$ of orientation-preserving Möbius transformations is isomorphic to $PSU(1,1)$. S and T are reflections, and thus orientation-reversing isometries, which are again $PSU(1,1)$ transformations, but of the form [16]

$$z \mapsto \gamma(z) = \frac{\alpha z^* + \beta}{\beta^* z^* + \alpha^*}, \tag{S31}$$

which is sometimes called an anti-Möbius or Möb$^-$ transformation, and is the composition of an ordinary Möbius transformation with complex conjugation. Together, Möb$^+$ and Möb$^-$ transformations form the general Möbius group, Möb, which is the full group Isom($\mathbb{H}$) of isometries of the hyperbolic plane. In the next subsections we figure out the explicit form of the generators R, S, T, U of G as $PSU(1,1)$ matrices. We first consider the Möb$^+$ generators R and U, then the Möb$^-$ generators S and T.

Transformation R. The transformation R is a C_8 rotation $z \mapsto R(z) = e^{i2\pi/8}z$ about the center of the octagonal unit cell (Fig. S2), which corresponds to the $SU(1,1)$ matrix

$$R = \begin{pmatrix} e^{i\pi/8} & 0 \\ 0 & e^{-i\pi/8} \end{pmatrix}, \tag{S32}$$

up to an overall sign.

Transformation U. This is a C_3 rotation around the center z_0 of a hyperbolic triangle, e.g., that formed by 0, λ, and $\lambda e^{i\pi/4}$ in Fig. S2, where $\lambda = \sqrt{\sqrt{2}-1}$ (see Sec. S1). The latter two vertices are found by considering that they are the midpoint of the boundary segments C_1 and C_2, respectively, which corresponds to the point $\theta = \pi$ in the parametrization (S1). The midpoints of C_1 and C_2 are thus $c - r$ and $e^{i\pi/4}(c-r)$, respectively, with c and r defined in Eq. (S2), and $c - r = \lambda$.

We first need to find the center z_0 of that triangle. By symmetry, we expect z_0 to lie on the straight geodesic (green line in Fig. S2) bisecting the triangle, i.e., $z_0 = ae^{i\pi/8}$ with $0 < a < \lambda$. We determine z_0 by requiring that it is the point of equal geodesic distance $d(z, z')$ from the three vertices of the triangle: $d(z_0, 0) = d(z_0, \lambda) = d(z_0, \lambda e^{i\pi/8})$. By symmetry, we see that the last equality is automatically satisfied, thus we only need to impose the first one to find a. The geodesic distance on the Poincaré disk is given by [16]

$$\cosh d(z, z') = 1 + \frac{2|z - z'|^2}{(1 - |z|^2)(1 - |z'|^2)}. \tag{S33}$$

Imposing the condition $d(z_0, z_1) = d(z_0, z_2)$ is thus the same as imposing

$$\frac{|z_0 - z_1|^2}{1 - |z_1|^2} = \frac{|z_0 - z_2|^2}{1 - |z_2|^2}. \tag{S34}$$

Setting $z_1 = 0$ and $z_2 = \lambda$, and after a bit of algebra, we obtain

$$a^2 - 2^{1/4}(1 + \sqrt{2})a + 1 = 0. \tag{S35}$$

Keeping the only root satisfying $0 < a < \lambda \approx 0.64$, we obtain

$$z_0 = \left(\frac{1 + \sqrt{2} - \sqrt{3}}{2^{3/4}}\right) e^{i\pi/8}. \tag{S36}$$

Now, U is a rotation by $2\pi/3$ around z_0. We do not directly know what this looks like, but we know that a rotation around $z = 0$ is $C_3 : z \mapsto e^{i2\pi/3}z$. To obtain U, we simply have to "translate" (boost) z_0 to the origin, perform C_3, then boost back to z_0. In other words, $U = \gamma_\eta \circ C_3 \circ \gamma_\eta^{-1}$ where γ_η is a boost from $z = 0$ to $z = z_0$, i.e., along the green geodesic joining $-e^{i\pi/8}$ to $e^{i\pi/8}$. To find γ_η, first consider a boost $\tilde{\gamma}_\eta$ along the x axis. Such a boost by a quantity in $-\infty < \eta < \infty$ is given by [16]

$$\tilde{\gamma}_\eta : z \mapsto \tilde{\gamma}_\eta(z) = \frac{(\cosh\eta)z + \sinh\eta}{(\sinh\eta)z + \cosh\eta}, \tag{S37}$$

corresponding to a $PSU(1,1)$ transformation with $\alpha = \cosh\eta$, $\beta = \sinh\eta$. The fixed points of this transformation are the points at infinity ± 1, and the origin is boosted to $\tilde{\gamma}_\eta(0) = \tanh\eta$. For $\eta > 0$ this is a boost towards the positive x axis. To boost along the green line, one should rotate down to the x axis, boost along the x axis, then rotate back to the green line. In other words, $\gamma_\eta = R_{\pi/8} \circ \tilde{\gamma}_\eta \circ R_{\pi/8}^{-1}$, where $R_{\pi/8} : z \mapsto e^{i\pi/8}z$ is a counterclockwise (C_{16}) rotation by $\pi/8$. We thus obtain

$$\gamma_\eta(z) = (R_{\pi/8} \circ \tilde{\gamma}_\eta \circ R_{\pi/8}^{-1})(z) = e^{i\pi/8}\left(\frac{(\cosh\eta)e^{-i\pi/8}z + \sinh\eta}{(\sinh\eta)e^{-i\pi/8}z + \cosh\eta}\right) = \frac{(\cosh\eta)z + e^{i\pi/8}\sinh\eta}{(e^{-i\pi/8}\sinh\eta)z + \cosh\eta}, \tag{S38}$$

a Möb$^+$ transformation with $\alpha = \cosh\eta$, $\beta = e^{i\pi/8}\sinh\eta$. The points at infinity $\pm e^{i\pi/8}$ are fixed points of this transformation.

We now need to find the value of η such that $\gamma_\eta(0) = z_0$. This implies

$$\tanh\eta = \frac{1+\sqrt{2}-\sqrt{3}}{2^{3/4}}. \tag{S39}$$

Writing $\sinh\eta = (1+\sqrt{2}-\sqrt{3})b$ and $\cosh\eta = 2^{3/4}b$, and finding b using $\cosh^2\eta - \sinh^2\eta = 1$, we find that γ_η is a Möb$^+$ transformation with

$$\alpha = \sqrt{\frac{\sqrt{3}+\sqrt{6}+3}{6}} > 1, \quad \beta = e^{i\pi/8}\sqrt{\alpha^2 - 1}. \tag{S40}$$

Finally, to obtain U we conjugate C_3 by γ_η. In $SU(1,1)$ this is simply a product of matrices, and we find that U is a Möb$^+$ transformation with

$$\alpha = e^{i3\pi/8}\sqrt{1+\frac{1}{\sqrt{2}}}, \quad \beta = 2^{-1/4}e^{-i3\pi/8}. \tag{S41}$$

Transformation S. The transformation S is defined in Ref. [33] as a reflection across the green line in Fig. S2. Define S' to be the reflection across the real axis, which sends $(x, y) \mapsto (x, -y)$ and is thus simply complex conjugation: $S' : z \mapsto z^*$, the simplest Möb$^-$ transformation. A reflection S across the green line can be obtained by first rotating by $\pi/8$ clockwise to the real axis, performing S', and then rotating back to the green line. We thus have:

$$S(z) = (R_{\pi/8} \circ S' \circ R^{-1}_{\pi/8})(z) = e^{i\pi/8}(e^{-i\pi/8}z)^* = e^{i2\pi/8}z^*, \tag{S42}$$

a transformation of the form (S31) with $\alpha = e^{i\pi/8}$, $\beta = 0$.

Transformation T. According to Ref. [33], T is a Möb$^-$ transformation which interchanges the origin $z = 0$ with a corner of the hyperbolic octagon, e.g., $p_1 = 2^{-1/4}e^{i\pi/8}$. Since it is a simple interchange, one should have $T^2 = e$ where e denotes the identity element in Möb$^+$. (Note that the composition of two Möb$^-$ transformations is in Möb$^+$, and the composition of a Möb$^+$ transformation and a Möb$^-$ transformation is a Möb$^-$ transformation.) Thus we have

$$T(z) = \frac{\alpha z^* + \beta}{\beta^* z^* + \alpha^*}, \tag{S43}$$

and wish to determine α and β. Imposing $T(0) = p_1$ implies $\beta = 2^{-1/4}e^{i\pi/8}\alpha^*$. Next, since $T^2 = e$ one has $T^2(0) = T(T(0)) = T(p_1) = 0$, which implies $\alpha e^{-i\pi/8} + \alpha^* e^{i\pi/8} = 0$. Since $\alpha \neq 0$, writing in polar form $\alpha = se^{i\theta}$, we find $\theta = 5\pi/8$. Finally, requiring that $|\alpha|^2 - |\beta|^2 = 1$, we find $s = 2^{1/4}\lambda^{-1} = \sqrt{2+\sqrt{2}}$. Thus we find that T is a transformation of the form (S31) with

$$\alpha = e^{i5\pi/8}\sqrt{2+\sqrt{2}}, \quad \beta = e^{-i\pi/2}\sqrt{1+\sqrt{2}}. \tag{S44}$$

Noting by explicit calculation that the composition $\gamma_1 \circ \gamma_2$ of two Möb$^-$ transformations γ_1, γ_2 with $SU(1,1)$ parameters α_1, β_1 and α_2, β_2 is a Möb$^+$ transformation with $SU(1,1)$ parameters α_{12}, β_{12} given by

$$\alpha_{12} = \alpha_1 \alpha_2^* + \beta_1 \beta_2, \quad \beta_{12} = \alpha_1 \beta_2^* + \beta_1 \alpha_2, \tag{S45}$$

we find that the $SU(1,1)$ parameters of T^2 are $\alpha_{T^2} = |\alpha|^2 + \beta^2 = 1$ and $\beta_{T^2} = \alpha(\beta + \beta^*) = 0$, and thus $T^2 = e$.

Generator relations. According to Ref. [33], the automorphism group of the Bolza surface admits a presentation with generators R, S, T, U and relations

$$R^8 = S^2 = T^2 = U^3 = (RS)^2 = (ST)^2 = RTR^3T = e, \tag{S46}$$

$$UR = R^7U^2, \tag{S47}$$

$$U^2R = STU, \tag{S48}$$

$$US = SU^2, \tag{S49}$$

$$UT = RSU. \tag{S50}$$

Using the explicit form of the generators just derived, we verify that all relations hold as identities in Möb, except $RTR^3T = e$. We find

$$RTR^3T = \gamma_3 \gamma_4^{-1} \gamma_1^{-1} \in \Gamma, \tag{S51}$$

where $\gamma_1, \gamma_2, \gamma_3, \gamma_4$ are the Fuchsian group generators (S4), thus RTR^3T indeed reduces to the identity in $G = \text{Aut}(\Sigma_2) \cong N(\Gamma)/\Gamma$. Similarly, Ref. [33] states that $Z(G)$, the center of G (i.e., the set of elements that commute with every element of G), is isomorphic to $\mathbb{Z}_2$ and generated by $R^4 : z \mapsto -z$. To verify this, it is sufficient to verify that R^4 commutes with S, T, U. Commutation with S holds as an identity in Möb, while for the remaining two generators we find

$$\gamma_1 R^4 U = UR^4, \quad \gamma_1 \gamma_4 \gamma_3^{-1} \gamma_2 R^4 T = TR^4, \tag{S52}$$

which indeed reduce to $R^4U = UR^4$ and $R^4T = TR^4$ in the quotient $G \cong N(\Gamma)/\Gamma$.

2. Point-group action on hyperbolic $\boldsymbol{k}$-space

In the previous section, we explicitly constructed the action of the point group G in real space. In this section, we determine how G acts in four-dimensional $\boldsymbol{k}$-space, via its action on the automorphic Bloch eigenstates.

Given a general (orientation-preserving or -reversing) transformation $\gamma \in$ Möb, define a linear operator $\mathcal{S}_\gamma$ which performs the corresponding transformation on a wavefunction $\psi(x, y) = \psi(z)$:

$$\mathcal{S}_\gamma \psi(z) \equiv \psi(\gamma(z)). \tag{S53}$$

For the derivation that follows, it will be useful to treat z and z^* as independent "real" variables, and the transformation (S53) will be written as $\mathcal{S}_\gamma\psi(z,z^*) = \psi(\gamma(z),\gamma(z)^*)$.

Point-group symmetries of the Hamiltonian. We assume the potential V is not only automorphic with respect to the Fuchsian group Γ of hyperbolic lattice translations, but also invariant under point-group transformations,

$$\mathcal{S}_h V(z,z^*)\mathcal{S}_h^{-1} = V(h(z),h(z)^*) = V(z,z^*), \qquad \forall h \in G \subset \text{Möb}\,. \tag{S54}$$

In other words, $[\mathcal{S}_h, V] = 0$ for all $h \in G$. We also want to show the kinetic term $H_0 = -\Delta$ commutes with $\mathcal{S}_h$, where

$$\Delta = (1-zz^*)^2\frac{\partial^2}{\partial z\partial z^*}, \tag{S55}$$

is the hyperbolic Laplacian [Eq. (1) in the main text], where we have used $\partial_x = \partial_z + \partial_{z^*}$ and $\partial_y = i(\partial_z - \partial_{z^*})$. We can in fact show that Δ is invariant under the action of any $\gamma \in$ Möb. First, Δ is obviously invariant under complex conjugation $z \mapsto z^*$. Since any Möb$^-$ transformation can be written as the composition of a Möb$^+$ transformation with complex conjugation, it is sufficient to show that Δ is invariant under Möb$^+$ transformations. Consider an arbitrary Möb$^+$ transformation,

$$z \mapsto w \equiv \gamma(z) = \frac{\alpha z + \beta}{\beta^* z + \alpha^*}, \qquad z^* \mapsto w^* \equiv \gamma(z)^* = \frac{\alpha^* z^* + \beta^*}{\beta z^* + \alpha}. \tag{S56}$$

We have

$$\begin{aligned}(\Delta\mathcal{S}_\gamma)\psi(z,z^*) &= (1-zz^*)^2\frac{\partial^2}{\partial z\partial z^*}\psi(w,w^*)\\ &= (1-zz^*)^2\frac{\partial w}{\partial z}\frac{\partial w^*}{\partial z^*}\frac{\partial^2}{\partial w\partial w^*}\psi(w,w^*)\\ &= (1-zz^*)^2\left|\frac{\partial w}{\partial z}\right|^2\frac{\partial^2}{\partial w\partial w^*}\psi(w,w^*).\end{aligned} \tag{S57}$$

On the other hand, we have

$$(\mathcal{S}_\gamma\Delta)\psi(z,z^*) = \mathcal{S}_\gamma\left((1-zz^*)^2\frac{\partial^2}{\partial z\partial z^*}\psi(z,z^*)\right) = (1-ww^*)^2\frac{\partial^2}{\partial w\partial w^*}\psi(w,w^*). \tag{S58}$$

Using (S56), we have

$$1 - ww^* = \frac{1-zz^*}{|\beta^* z + \alpha^*|^2}. \tag{S59}$$

On the other hand, we have

$$\frac{\partial w}{\partial z} = \frac{1}{(\beta^* z + \alpha^*)^2}, \tag{S60}$$

thus $[\mathcal{S}_\gamma, \Delta] = 0$ for any $\gamma \in$ Möb, and in particular for $\gamma \in G$. As a result, $[\mathcal{S}_h, H] = 0$ for any $h \in G$, with $H = -\Delta + V$ the full Hamiltonian.

Point-group symmetries of hyperbolic Bloch eigenstates. We now go back to treating $z = x+iy \in \mathbb{C}$ as a complex coordinate in the Poincaré disk. Consider an eigenstate $\psi_{\boldsymbol{k}}(z)$ of H with energy $E(\boldsymbol{k})$, that obeys the four automorphic Bloch conditions (S13):

$$\psi_{\boldsymbol{k}}(\gamma_j(z)) = e^{ik_j}\psi_{\boldsymbol{k}}(z), \quad j = 1, \ldots, 4. \tag{S61}$$

with $\gamma_1, \ldots, \gamma_4$ in Eq. (S4). Since $\mathcal{S}_h$ with $h \in G$ commutes with H, the state

$$\psi_{\boldsymbol{k}}^h(z) \equiv \mathcal{S}_h \psi_{\boldsymbol{k}}(z) = \psi_{\boldsymbol{k}}(h(z)), \tag{S62}$$

for a given $h \in G$ is also an eigenstate of H with the same eigenenergy $E(\boldsymbol{k})$. However, it does not in general obey the same Bloch conditions as $\psi_{\boldsymbol{k}}(z)$. Indeed, first write Eq. (S61) as

$$\mathcal{S}_{\gamma_j}\psi_{\boldsymbol{k}}(z) = e^{ik_j}\psi_{\boldsymbol{k}}(z), \quad j = 1, \ldots, 4. \tag{S63}$$

Acting with $\mathcal{S}_h$ on both sides and inserting the identity in the form $\mathcal{S}_h^{-1}\mathcal{S}_h = \mathbb{I}$, where the defining action of the inverse operator is

$$\mathcal{S}_\gamma^{-1}\psi(z) = \psi(\gamma^{-1}(z)), \quad \gamma \in \text{Möb}, \tag{S64}$$

and $\mathbb{I}$ is the identity operator, we have

$$\mathcal{S}_h \mathcal{S}_{\gamma_j} \mathcal{S}_h^{-1} \psi_{\boldsymbol{k}}^h(z) = e^{ik_j}\psi_{\boldsymbol{k}}^h(z), \quad j = 1, \ldots, 4. \tag{S65}$$

In other words, $\psi_{\boldsymbol{k}}^h$ obeys the modified Bloch conditions

$$\psi_{\boldsymbol{k}}^h((h\gamma_j h^{-1})(z)) = e^{ik_j}\psi_{\boldsymbol{k}}^h(z), \quad j = 1, \ldots, 4. \tag{S66}$$

The modified boundary conditions (S66) involve the conjugation of the Fuchsian group generator $\gamma_j \in \Gamma$ by point-group elements $h \in G$. Given Eqs. (S26-S27), $h\gamma_j h^{-1}$ is again necessarily in Γ. (Although h can be either in Möb$^+$ or Möb$^-$, $h\gamma_j h^{-1}$ is necessarily in Möb$^+$, since the inverse of an element of Möb$^-$ is also in Möb$^-$, and only an even number (which could be zero) of Möb$^-$ transformations appear in $h\gamma_j h^{-1}$. Therefore the boundary conditions obeyed by $\psi_{\boldsymbol{k}}^h$ preserve orientation, as those for $\psi_{\boldsymbol{k}}$.) From the explicit forms of the four generators $h = R, S, T, U$ of G, determined earlier, and the four generators γ_j in Eq. (S4), we can compute $h\gamma_j h^{-1}$. We display the result of these computations in Table S1, with $h \in \{R, S, T, U\}$ as the row index and $\gamma_j \in \{\gamma_1, \gamma_2, \gamma_3, \gamma_4\}$ as the column index. These expressions in terms of the γ_j are not unique, since one can use the relation (S6) to obtain different (but equivalent) expressions. Furthermore, since $h\gamma_j^{-1}h^{-1} = (h\gamma_j h^{-1})^{-1}$, the conjugated inverse generators $h\gamma_j^{-1}h^{-1}$ are easily determined from Table S1 as well.

	γ_1	γ_2	γ_3	γ_4
R	γ_2	γ_3	γ_4	γ_1^{-1}
S	γ_2	γ_1	γ_4^{-1}	γ_3^{-1}
T	$\gamma_4^{-1}\gamma_3\gamma_2^{-1}$	$\gamma_3\gamma_4^{-1}\gamma_1^{-1}$	$\gamma_2\gamma_4^{-1}\gamma_1^{-1}$	$\gamma_2\gamma_3^{-1}\gamma_1^{-1}$
U	$\gamma_2\gamma_1^{-1}$	γ_1^{-1}	$\gamma_4^{-1}\gamma_1^{-1}$	$\gamma_4^{-1}\gamma_3\gamma_2^{-1}$

TABLE S1. Conjugation of Fuchsian group generators by point-group symmetries.

Using the results above, we can now show that $\psi_{\boldsymbol{k}}^h$ corresponds to a hyperbolic Bloch eigenstate with a transformed wavevector $\boldsymbol{k}^h$, i.e.,

$$\psi_{\boldsymbol{k}}^h(\gamma_j(z)) = e^{ik_j^h}\psi_{\boldsymbol{k}}^h(z), \quad j = 1, \dots, 4. \tag{S67}$$

To show this, we first observe that for a given point-group generator h, the relations in Table S1 can be inverted to express any original generator γ_j in terms of the conjugated generators $\gamma_j^h \equiv h\gamma_j h^{-1}$. We find

$$\gamma_1 = (\gamma_4^R)^{-1} = \gamma_2^S = (\gamma_4^T)^{-1}\gamma_3^T(\gamma_2^T)^{-1} = (\gamma_2^U)^{-1}, \tag{S68}$$
$$\gamma_2 = \gamma_1^R = \gamma_1^S = \gamma_3^T(\gamma_4^T)^{-1}(\gamma_1^T)^{-1} = \gamma_1^U(\gamma_2^U)^{-1}, \tag{S69}$$
$$\gamma_3 = \gamma_2^R = (\gamma_4^S)^{-1} = \gamma_2^T(\gamma_4^T)^{-1}(\gamma_1^T)^{-1} = \gamma_1^U\gamma_4^U(\gamma_3^U)^{-1}, \tag{S70}$$
$$\gamma_4 = \gamma_3^R = (\gamma_3^S)^{-1} = \gamma_2^T(\gamma_3^T)^{-1}(\gamma_1^T)^{-1} = \gamma_2^U(\gamma_3^U)^{-1}. \tag{S71}$$

Writing Eq. (S66) as

$$\psi_{\boldsymbol{k}}^h(\gamma_j^h(z)) = e^{ik_j}\psi_{\boldsymbol{k}}^h(z), \quad j = 1, \dots, 4, \tag{S72}$$

we have, taking $h = R$,

$$\psi_{\boldsymbol{k}}^R(\gamma_1(z)) = \psi_{\boldsymbol{k}}^R((\gamma_4^R)^{-1}(z)) = e^{-ik_4}\psi_{\boldsymbol{k}}^R(z), \tag{S73}$$
$$\psi_{\boldsymbol{k}}^R(\gamma_2(z)) = \psi_{\boldsymbol{k}}^R(\gamma_1^R(z)) = e^{ik_1}\psi_{\boldsymbol{k}}^R(z), \tag{S74}$$
$$\psi_{\boldsymbol{k}}^R(\gamma_3(z)) = \psi_{\boldsymbol{k}}^R(\gamma_2^R(z)) = e^{ik_2}\psi_{\boldsymbol{k}}^R(z), \tag{S75}$$
$$\psi_{\boldsymbol{k}}^R(\gamma_4(z)) = \psi_{\boldsymbol{k}}^R(\gamma_3^R(z)) = e^{ik_3}\psi_{\boldsymbol{k}}^R(z), \tag{S76}$$

where in Eq. (S73) we have used the relation $\psi_{\boldsymbol{k}}^h((\gamma_j^h)^{-1}(z)) = e^{-ik_j}\psi_{\boldsymbol{k}}^h(z)$, $j = 1, \dots, 4$, easily shown by substituting $z \to (\gamma_j^h)^{-1}(z)$ in Eq. (S72). Thus Eqs. (S73-S76) can be written as Eq. (S67) with

$$\boldsymbol{k}^R = (k_1^R, k_2^R, k_3^R, k_4^R) = (-k_4, k_1, k_2, k_3). \tag{S77}$$

Proceeding similarly for $h = S, T, U$, we find that the $\psi_{\boldsymbol{k}}^h$ obey Eq. (S67) with

$$\boldsymbol{k}^S = (k_1^S, k_2^S, k_3^S, k_4^S) = (k_2, k_1, -k_4, -k_3), \tag{S78}$$
$$\boldsymbol{k}^T = (k_1^T, k_2^T, k_3^T, k_4^T) = (-k_2 + k_3 - k_4, -k_1 + k_3 - k_4, -k_1 + k_2 - k_4, -k_1 + k_2 - k_3), \tag{S79}$$
$$\boldsymbol{k}^U = (k_1^U, k_2^U, k_3^U, k_4^U) = (-k_2, k_1 - k_2, k_1 - k_3 + k_4, k_2 - k_3). \tag{S80}$$

The linear relations (S77-S80) can be written in the matrix form of Eq. (S28),

$$k_i^h = M_{ij}(h)k_j, \tag{S81}$$

with the 4×4 matrices:

$$\begin{aligned} M(R) &= \begin{pmatrix} 0 & 0 & 0 & -1 \\ 1 & 0 & 0 & 0 \\ 0 & 1 & 0 & 0 \\ 0 & 0 & 1 & 0 \end{pmatrix}, \qquad M(S) = \begin{pmatrix} 0 & 1 & 0 & 0 \\ 1 & 0 & 0 & 0 \\ 0 & 0 & 0 & -1 \\ 0 & 0 & -1 & 0 \end{pmatrix}, \\ M(T) &= \begin{pmatrix} 0 & -1 & 1 & -1 \\ -1 & 0 & 1 & -1 \\ -1 & 1 & 0 & -1 \\ -1 & 1 & -1 & 0 \end{pmatrix}, \; M(U) = \begin{pmatrix} 0 & -1 & 0 & 0 \\ 1 & -1 & 0 & 0 \\ 1 & 0 & -1 & 1 \\ 0 & 1 & -1 & 0 \end{pmatrix}. \end{aligned} \tag{S82}$$

Equation (S81) can be thought of as the genus-2 analog of the Euclidean relation $k_i^h = h_{ij}k_j$, that follows from straightforward Fourier analysis, where $h \in G \subset O(2)$ is a Euclidean point-group transformation $r_i \to h_{ij}r_j$ in real space. In fact, we find that the matrices (S82) form a linear representation of the automorphism group G of the Bolza surface in four-dimensional hyperbolic $\boldsymbol{k}$-space, in the sense that those matrices obey the group relations (S46-S50), with $M(h_1h_2) = M(h_1)M(h_2)$ for $h_1, h_2 \in G$, and $M(e)$ is the 4×4 identity matrix.

S5. THE TIGHT-BINDING APPROXIMATION

We now discuss how an analog of the tight-binding approximation [2] can be devised within our general hyperbolic band theory. Consider first the quantum mechanics of an isolated, deep potential well $U(z)$ with compact support in $\mathcal{D}$, e.g., the circular well considered in the main text with V_0 large. As with other potentials (e.g., Ref. [49]), we expect a number of bound states with discrete eigenenergies ϵ_n and localized wavefunctions $\phi_n(z)$, orthonormal with respect to the Poincaré metric, and satisfying an atomic-like Schrödinger equation:

$$(-\Delta + U(z))\phi_n(z) = \epsilon_n\phi_n(z), \qquad z \in \mathbb{H}. \tag{S83}$$

If the ϕ_n are sufficiently localized, they and their Γ-translates $\phi_n(\gamma(z))$, $\gamma \in \Gamma$, should be good approximations to the true wavefunctions of the full hyperbolic lattice with automorphic potential $V(z) = V(\gamma(z))$ [Eq. (5) in the main text]. To construct an approximate eigenstate that obeys the automorphic Bloch condition $\psi(\gamma(z)) = \chi(\gamma)\psi(z)$ with hyperbolic crystal momentum $\boldsymbol{k}$, we take an appropriate linear combination of those localized wavefunctions,

$$\psi_{\boldsymbol{k}}(z) \approx \sum_{\gamma\in\Gamma}\sum_n b_{n\boldsymbol{k}}\chi_{\boldsymbol{k}}^*(\gamma)\phi_n(\gamma(z)), \tag{S84}$$

where n ranges over the discrete levels of the atomic problem (S83), $b_{n\boldsymbol{k}}$ is an expansion coefficient, and we explicitly indicated by a subscript the dependence of the Bloch phase factor (S11) on $\boldsymbol{k}$. Substituting this approximate expansion into the full Schrödinger equation $(-\Delta+V)\psi_{\boldsymbol{k}} = E(\boldsymbol{k})\psi_{\boldsymbol{k}}$, multiplying from the left on both sides by $\phi_m^*(z)$, and integrating over the entire Poincaré disk, we obtain a hyperbolic analog of the standard tight-binding equations [2]:

$$\sum_n \left(\epsilon_m s_{mn}(\boldsymbol{k}) - u_{mn} - t_{mn}(\boldsymbol{k})\right) b_{n\boldsymbol{k}} \approx E(\boldsymbol{k}) \sum_n s_{mn}(\boldsymbol{k}) b_{n\boldsymbol{k}}, \tag{S85}$$

a generalized matrix eigenvalue problem whose eigenvalues are approximate hyperbolic band energies $\{E_n(\boldsymbol{k})\}$ and whose eigenvectors are the expansion coefficients $b_{n\boldsymbol{k}}$. We define the overlap matrix $s_{mn}(\boldsymbol{k})$, the on-site potential matrix u_{mn}, and the hopping matrix $t_{mn}(\boldsymbol{k})$ as

$$s_{mn}(\boldsymbol{k}) = \delta_{mn} + \sum_{\gamma\neq e} \chi_{\boldsymbol{k}}^*(\gamma) \int_{\mathbb{H}} d^2z\sqrt{g}\phi_m^*(z)\phi_n(\gamma(z)), \tag{S86}$$

$$u_{mn} = -\int_{\mathbb{H}} d^2z\sqrt{g}\phi_m^*(z)\Delta V(z)\phi_n(z), \tag{S87}$$

$$t_{mn}(\boldsymbol{k}) = -\sum_{\gamma\neq e} \chi_{\boldsymbol{k}}^*(\gamma) \int_{\mathbb{H}} d^2z\sqrt{g}\phi_m^*(z)\Delta V(z)\phi_n(\gamma(z)), \tag{S88}$$

and $\Delta V = \sum_{\gamma\neq e} U(\gamma(z))$ is the sum of Γ-translates of $U(z)$, excluding the reference cell $\mathcal{D}$.

S6. HYPERBOLIC WANNIER FUNCTIONS AND THE BLOCH TRANSFORM

Another key notion of conventional band theory, conceptually related to the tight-binding approximation, is that of Wannier functions [2]. In the Euclidean context, these are defined as the exact Fourier coefficients $f_n(\boldsymbol{R}, \boldsymbol{r})$ of a true Bloch eigenstate $\psi_{n\boldsymbol{k}}(\boldsymbol{r})$, expanded for each $\boldsymbol{r}$ as a Fourier series in $\boldsymbol{k}$, with $\boldsymbol{R} \in \mathbb{Z}^2$ the sites of the real-space lattice:

$$f_n(\boldsymbol{r} - \boldsymbol{R}) = \int_{1\text{BZ}} \frac{d^2k}{(2\pi)^2} e^{-i\boldsymbol{k}\cdot\boldsymbol{R}} \psi_{n\boldsymbol{k}}(\boldsymbol{r}), \tag{S89}$$

where 1BZ denotes the Brillouin zone torus. Since $\psi_{n\boldsymbol{k}}(\boldsymbol{r})$ satisfies the Bloch condition, Wannier functions are invariant under simultaneous translations of $\boldsymbol{r}$ and $\boldsymbol{R}$ by a given lattice vector $\boldsymbol{m} \in \mathbb{Z}^2$, and are thus necessarily of the form $f_n(\boldsymbol{r} - \boldsymbol{R})$. They can be interpreted as atomic-like wavefunctions associated with site $\boldsymbol{R}$, analogous to the atomic orbitals. Viewed as a function of $\boldsymbol{r} \in \mathbb{R}^2$, a Euclidean Wannier function $f_n(\boldsymbol{r} - \boldsymbol{R})$ is localized around $\boldsymbol{R}$, and therefore lives in the space $L^2(\mathbb{R}^2)$ of square-integrable functions over the Euclidean plane $\mathbb{R}^2$. Standard Fourier analysis allows us to invert Eq. (S89):

$$\psi_{n\boldsymbol{k}}(\boldsymbol{r}) = \sum_{\boldsymbol{R}} e^{i\boldsymbol{k}\cdot\boldsymbol{R}} f_n(\boldsymbol{r} - \boldsymbol{R}). \tag{S90}$$

We will refer to Eq. (S90) as the (Euclidean) *Bloch transform*, and to Eq. (S89) as the *inverse Bloch transform*.

The tight-binding approximation (S84) was presented as a means to obtain a delocalized wavefunction $\psi_{\boldsymbol{k}}(z)$ obeying the automorphic Bloch condition on $z \in \mathbb{H}$ from a given localized wavefunction $\phi_n(z)$ and its Γ-translates $\phi_n(\gamma(z))$. However, we may ask whether such a representation can hold *exactly*, i.e., whether one can devise a precise hyperbolic analog of the Bloch transform and its inverse. The natural generalization of the Euclidean inverse Bloch transform (S89) would be:

$$f_n(\gamma(z)) \stackrel{?}{=} \int_{\mathrm{Jac}(\Sigma_g)} \frac{d^{2g}k}{(2\pi)^{2g}} \chi_{\boldsymbol{k}}(\gamma)\psi_{n\boldsymbol{k}}(z), \tag{S91}$$

where we consider the general case of a $\{4g, 4g\}$ tessellation with $g \geqslant 2$. By assumption, $\psi_{n\boldsymbol{k}}(z)$ obeys the automorphic Bloch condition, thus the putative hyperbolic Wannier function $f_n(\gamma(z))$ depends only on $\gamma(z)$, and not γ and z separately. This mirrors the behavior $f_n(\boldsymbol{R}, \boldsymbol{r}) = f_n(\boldsymbol{r} - \boldsymbol{R})$ of Euclidean Wannier functions.

However, we find that $f_n(\gamma\gamma^{(1)}(z)) = f_n(\gamma(z))$ for any $\gamma^{(1)} \in \Gamma^{(1)}$ where

$$\Gamma^{(1)} = [\Gamma, \Gamma] = \langle \gamma_i\gamma_j\gamma_i^{-1}\gamma_j^{-1} \rangle, \quad i, j = 1, \ldots, 2g, \tag{S92}$$

is the commutator subgroup of $\Gamma \cong \pi_1(\Sigma_g)$, and $\gamma_1, \ldots, \gamma_{2g}$ are the $2g$ generators of Γ. Loosely speaking, the commutator subgroup of a group measures the extent to which the group is non-abelian (the commutator subgroup of an abelian group is the trivial group with only the identity element). The $\Gamma^{(1)}$-periodicity of $f_n(\gamma(z))$ follows from the fact that $\chi_{\boldsymbol{k}}(\gamma\gamma^{(1)}) = \chi_{\boldsymbol{k}}(\gamma)$, i.e., because $\Gamma^{(1)}$ is in the kernel of the representation $\chi_{\boldsymbol{k}} : \Gamma \to U(1)$ for all $\boldsymbol{k} \in \mathrm{Jac}(\Sigma_g)$. In other words, the space of functions $\psi_{n\boldsymbol{k}}$ obeying the automorphic Bloch condition only contains functions that are $\Gamma^{(1)}$-invariant. As a result, any attempt to construct localized Wannier functions $f_n(\gamma(z))$ from such automorphic Bloch functions can only yield functions that are at most $\Gamma^{(1)}$-invariant. Since $\Gamma^{(1)}$ is an infinite group, $\Gamma^{(1)}$-invariant functions cannot be localized and are not square integrable (i.e., they are not part of $L^2(\mathbb{H})$, except the trivial function $f_n = 0$). This is problematic for the construction of a naive hyperbolic analog of the Bloch transform (S90). Indeed, one would write:

$$\psi_{n\boldsymbol{k}}(z) \stackrel{?}{=} \sum_{\gamma\in\Gamma} \chi_{\boldsymbol{k}}^*(\gamma) f_n(\gamma(z)) = \left(\mathrm{vol}\,\Gamma^{(1)}\right) \sum_{[\gamma]\in\Gamma/\Gamma^{(1)}} \chi_{\boldsymbol{k}}^*([\gamma]) f_n([\gamma](z)), \tag{S93}$$

where $\mathrm{vol}\,\Gamma^{(1)}$ denotes the volume of the group $\Gamma^{(1)}$, and we have used the fact that $\chi_{\boldsymbol{k}}(\gamma)$ and $f_n(\gamma(z))$ are both $\Gamma^{(1)}$-periodic, and thus depend only on the coset $[\gamma]$ of $\Gamma^{(1)}$ in Γ to which γ belongs (since $\Gamma^{(1)}$ is normal in Γ, the choice of left or right coset is immaterial). Since $\mathrm{vol}\,\Gamma^{(1)}$ is infinite, the sum over $\gamma \in \Gamma$ in the naive hyperbolic Bloch transform (S93) cannot possibly converge.

The appearance of the troublesome infinite factor $\mathrm{vol}\,\Gamma^{(1)}$ suggests an obvious resolution to the problem, namely that we define hyperbolic Wannier functions as the set $\{f_n([\gamma](z)) : [\gamma] \in \Gamma/\Gamma^{(1)}\}$,

in which case the Bloch transform becomes:

$$\psi_{n\boldsymbol{k}}(z) = \sum_{[\gamma]\in\Gamma/\Gamma^{(1)}} \chi^*_{\boldsymbol{k}}([\gamma]) f_n([\gamma](z)). \tag{S94}$$

Since $\Gamma/\Gamma^{(1)}$ is nothing but the abelianization of $\Gamma \cong \pi_1(\Sigma_g)$, which is isomorphic to the first homology group $H_1(\Sigma_g, \mathbb{Z}) \cong \mathbb{Z}^{2g}$, the sum in Eq. (S94) is in fact over the sites $\boldsymbol{R} \in \mathbb{Z}^{2g}$ of a $2g$-dimensional Euclidean lattice. We finally recover standard Fourier analysis, albeit in higher dimensions, with the inverse Bloch transform given by:

$$f_n([\gamma](z)) = \int_{\mathrm{Jac}(\Sigma_g)} \frac{d^{2g}k}{(2\pi)^{2g}} \chi_{\boldsymbol{k}}([\gamma]) \psi_{n\boldsymbol{k}}(z). \tag{S95}$$

Indeed, viewed as functions of $\boldsymbol{k} \in \mathrm{Jac}(\Sigma_g)$, the characters $\chi_{\boldsymbol{k}}([\gamma]) = e^{i\boldsymbol{k}\cdot\boldsymbol{R}}$ are orthonormal:

$$\int_{\mathrm{Jac}(\Sigma_g)} \frac{d^{2g}k}{(2\pi)^{2g}} \chi_{\boldsymbol{k}}([\gamma]) \chi^*_{\boldsymbol{k}}([\gamma']) = \delta_{[\gamma],[\gamma']}, \qquad [\gamma], [\gamma'] \in H_1(\Sigma_g, \mathbb{Z}) \cong \mathbb{Z}^{2g}. \tag{S96}$$

Note that we also retain the property that the hyperbolic Wannier functions (S95) depend only on $[\gamma](z)$, as opposed to depending on $[\gamma]$ and z separately. Viewed from this more abstract point of view, the sum over lattice sites $\boldsymbol{R} \in \mathbb{Z}^2$ in the Euclidean Bloch transform (S90) is in fact over the elements of $H_1(\Sigma_1, \mathbb{Z}) \cong \mathbb{Z}^2$, where $\Sigma_1 \cong T^2$ is the compactified toroidal unit cell. For a Euclidean lattice, the commutator subgroup is trivial, thus the fundamental group $\pi_1(\Sigma_1)$ and first homology group $H_1(\Sigma_1, \mathbb{Z})$ are isomorphic (and abelian).

When considering the hyperbolic Wannier functions (S95) as functions of $z \in \mathbb{H}$, they are still $\Gamma^{(1)}$-periodic, and thus not in $L^2(\mathbb{H})$. However, they become localized on the quotient $\mathbb{H}/\Gamma^{(1)}$, which is an infinite-sheeted abelian cover of Σ_g. This can be considered as an infinite subset of the original tessellation on which the hyperbolic Bloch states $\psi(\gamma(z)) = \chi(\gamma)\psi(z)$ form a complete set; the corresponding Wannier functions $f_n([\gamma](z))$ are then finally in $L^2(\mathbb{H}/\Gamma^{(1)})$. The group of translations on $\mathbb{H}/\Gamma^{(1)}$ is now the abelian group $\Gamma/\Gamma^{(1)} \cong H_1(\Sigma_g, \mathbb{Z}) \cong \mathbb{Z}^{2g}$. There exists a family of translation operators $\{T_{[\gamma]} : [\gamma] \in \Gamma/\Gamma^{(1)}\}$ acting on $L^2(\mathbb{H}/\Gamma^{(1)})$ which forms a mutually commuting set that can be simultaneously diagonalized, with eigenvalues $\chi_{\boldsymbol{k}}([\gamma])$ corresponding to a complete set of unitary irreducible representations of $\Gamma/\Gamma^{(1)}$.

To complete our discussion of hyperbolic Wannier functions, we show that they obey the expected property [2] of being orthonormal for different bands n, n' and different homology classes $[\gamma], [\gamma'] \in H_1(\Sigma_g, \mathbb{Z})$. The inner product of two hyperbolic Wannier functions on $\mathbb{H}/\Gamma^{(1)}$ is:

$$\int_{\mathbb{H}/\Gamma^{(1)}} d^2z\sqrt{g} f^*_n([\gamma](z)) f_{n'}([\gamma'](z)) = \int_{\mathrm{Jac}(\Sigma_g)} \frac{d^{2g}k}{(2\pi)^{2g}} \int_{\mathrm{Jac}(\Sigma_g)} \frac{d^{2g}k'}{(2\pi)^{2g}} \chi^*_{\boldsymbol{k}}([\gamma]) \chi_{\boldsymbol{k}'}([\gamma'])$$
$$\times \int_{\mathbb{H}/\Gamma^{(1)}} d^2z\sqrt{g}\, \psi^*_{n\boldsymbol{k}}(z) \psi_{n'\boldsymbol{k}'}(z). \tag{S97}$$

Here $\sqrt{g}$ denotes the square root of the determinant of the Poincaré metric (not to be confused with the genus). For $\boldsymbol{k} \neq \boldsymbol{k}'$, the hyperbolic Bloch eigenstates $\psi_{n\boldsymbol{k}}(z)$ and $\psi_{n'\boldsymbol{k}'}(z)$, as constructed

in our work, obey different boundary conditions and thus formally live in different Hilbert spaces; we cannot directly invoke their orthogonality. To circumvent this problem, we use the invariance of the integration measure $d^2z\sqrt{g}$ under Möbius transformations and the fact that $\mathcal{D}$ is a fundamental region for Γ to write

$$\mathbb{H}/\Gamma^{(1)} = \bigsqcup_{[\gamma]\in\Gamma/\Gamma^{(1)}} [\gamma](\mathcal{D}), \tag{S98}$$

where $\sqcup$ denotes disjoint union, and thus

$$\begin{aligned}\int_{\mathbb{H}/\Gamma^{(1)}} d^2z\sqrt{g}\psi^*_{n\boldsymbol{k}}(z)\psi_{n'\boldsymbol{k}'}(z) &= \sum_{[\gamma]\in\Gamma/\Gamma^{(1)}} \int_{\mathcal{D}} d^2z\sqrt{g}\psi^*_{n\boldsymbol{k}}([\gamma](z))\psi_{n'\boldsymbol{k}'}([\gamma](z)) \\ &= \sum_{[\gamma]\in\Gamma/\Gamma^{(1)}} \chi^*_{\boldsymbol{k}}([\gamma])\chi_{\boldsymbol{k}'}([\gamma]) \int_{\mathcal{D}} d^2z\sqrt{g}\psi^*_{n\boldsymbol{k}}(z)\psi_{n'\boldsymbol{k}'}(z),\end{aligned} \tag{S99}$$

using the automorphic Bloch condition. Equation (S98) follows from the fact that the Fuchsian group Γ acts properly discontinuously on $\mathbb{H}$ [17], thus $\mathcal{D}\cap\gamma(\mathcal{D}) = 0$ for any $\gamma \in \Gamma\backslash\{e\}$. The Bloch characters $\chi_{\boldsymbol{k}}([\gamma]) = e^{i\boldsymbol{k}\cdot\boldsymbol{R}}$, $\boldsymbol{R} \in \Gamma/\Gamma^{(1)} \cong \mathbb{Z}^{2g}$ obey a Schur-type orthogonality relation familiar from Fourier analysis:

$$\sum_{[\gamma]\in\Gamma/\Gamma^{(1)}} \chi^*_{\boldsymbol{k}}([\gamma])\chi_{\boldsymbol{k}'}([\gamma]) = \sum_{\boldsymbol{R}\in\mathbb{Z}^{2g}} e^{-i(\boldsymbol{k}-\boldsymbol{k}')\cdot\boldsymbol{R}} = (2\pi)^{2g}\delta(\boldsymbol{k}-\boldsymbol{k}'). \tag{S100}$$

Substituting into Eq. (S99), we have

$$\int_{\mathbb{H}/\Gamma^{(1)}} d^2z\sqrt{g}\psi^*_{n\boldsymbol{k}}(z)\psi_{n'\boldsymbol{k}'}(z) = (2\pi)^{2g}\delta(\boldsymbol{k}-\boldsymbol{k}') \int_{\mathcal{D}} d^2z\sqrt{g}\psi^*_{n\boldsymbol{k}}(z)\psi_{n'\boldsymbol{k}}(z) = (2\pi)^{2g}\delta(\boldsymbol{k}-\boldsymbol{k}')\delta_{nn'}, \tag{S101}$$

using the orthogonality of hyperbolic Bloch eigenstates on $\mathcal{D}$ with the same $\boldsymbol{k}$. Substituting in Eq. (S97), we finally obtain:

$$\int_{\mathbb{H}/\Gamma^{(1)}} d^2z\sqrt{g}f^*_n([\gamma](z))f_{n'}([\gamma'](z)) = \delta_{nn'} \int_{\mathrm{Jac}(\Sigma_g)} \frac{d^{2g}k}{(2\pi)^{2g}} \chi^*_{\boldsymbol{k}}([\gamma])\chi_{\boldsymbol{k}}([\gamma']) = \delta_{nn'}\delta_{[\gamma],[\gamma']}, \tag{S102}$$

making use of Eq. (S96) in the last equality. Thus the hyperbolic Wannier functions behave as Euclidean Wannier functions in $2g$ dimensions.

REFERENCES AND NOTES

1. F. Bloch, Über die Quantenmechanik der Elektronen in Kristallgittern. *Z. Phys.* **52**, 555–600 (1929).
2. N. W. Ashcroft, N. D. Mermin, *Solid State Physics* (Saunders College, 1976).
3. F. D. M. Haldane, Model for a quantum Hall effect without Landau levels: Condensed-matter realization of the "parity anomaly". *Phys. Rev. Lett.* **61**, 2015–2018 (1988).
4. C.-K. Chiu, J. C. Y. Teo, A. P. Schnyder, S. Ryu, Classification of topological quantum matter with symmetries. *Rev. Mod. Phys.* **88**, 035005 (2016).
5. P. J. Steinhardt, S. Ostlund, *The Physics of Quasicrystals* (World Scientific, 1987).
6. T. Janssen, A. Janner, Aperiodic crystals and superspace concepts. *Acta Crystallogr. B Struct. Sci. Cryst. Eng. Mater.* **70**, 617–651 (2014).
7. A. J. Kollár, M. Fitzpatrick, A. A. Houck, Hyperbolic lattices in circuit quantum electrodynamics. *Nature* **571**, 45–50 (2019).
8. H. S. M. Coxeter, Crystal symmetry and its generalizations. *Royal Soc. Canada* **51**, 1–13 (1957).
9. H. S. M. Coxeter, The non-Euclidean symmetry of Escher's picture 'Circle Limit III'. *Leonardo* **12**, 19–25 (1979).
10. A. J. Kollár, M. Fitzpatrick, P. Sarnak, A. A. Houck, Line-graph lattices: Euclidean and non-euclidean flat bands, and implementations in circuit quantum electrodynamics. *Commun. Math. Phys.* **376**, 1909–1956 (2020).
11. I. Boettcher, P. Bienias, R. Belyansky, A. J. Kollár, A. V. Gorshkov, Quantum simulation of hyperbolic space with circuit quantum electrodynamics: From graphs to geometry. *Phys. Rev. A* **102**, 032208 (2020).
12. S. Yu, X. Piao, N. Park, Topological hyperbolic lattices. *Phys. Rev. Lett.* **125**, 053901 (2020).
13. D. Mumford, *Tata Lectures on Theta I* (Birkhäuser, 1983).
14. M. Nakahara, *Geometry, Topology, and Physics* (IOP Publishing, 1990).
15. J. H. Silverman, Elliptic curves and cryptography. *Proc. Sympos. Appl. Math.* **62**, 91–112 (2005).
16. N. L. Balazs, A. Voros, Chaos on the pseudosphere. *Phys. Rep.* **143**, 109–240 (1986).
17. S. Katok, *Fuchsian Groups* (The University of Chicago Press, 1992).
18. H. M. Farkas, I. Kra, *Riemann Surfaces* (Springer, ed. 2, 1992).
19. R. C. Gunning, The structure of factors of automorphy. *Amer. J. Math.* **78**, 357–382 (1956).
20. A. B. Venkov, *Spectral Theory of Automorphic Functions and Its Applications* (Kluwer Academic Publishers, 1990).

21. D. A. Hejhal, *The Selberg Trace Formula for* $PSL(2,\mathbb{R})$, *Volume 1* (Springer-Verlag, 1976).
22. G. Kiremidjian, Complex structures on Riemann surfaces. *Trans. Amer. Math. Soc.* **169**, 317–336 (1972).
23. O. Forster, *Lectures on Riemann Surfaces* (Springer-Verlag, 1981).
24. R. Miranda, *Algebraic Curves and Riemann Surfaces* (American Mathematical Society, 1995).
25. I. G. Macdonald, Symmetric products of an algebraic curve. *Topology* **1**, 319–343 (1962).
26. P. Buser, *Geometry and Spectra of Compact Riemann Surfaces* (Birkhäuser, 1992).
27. R. Aurich, F. Steiner, Periodic-orbit sum rules for the Hadamard-Gutzwiller model. *Physica D* **39**, 169–193 (1989).
28. R. Aurich, F. Steiner, Statistical properties of highly excited quantum eigenstates of a strongly chaotic system. *Physica D* **64**, 185–214 (1993).
29. H. Ninnemann, Gutzwiller's octagon and the triangular billiard $T^*(2,3,8)$ as models for the quantization of chaotic systems by Selberg's trace formula. *Int. J. Mod. Phys. B* **9**, 1647–1753 (1995).
30. A. Bachelot-Motet, Wave computation on the hyperbolic double doughnut. *J. Comput. Math.* **28**, 790–806 (2010).
31. A. Strohmaier, V. Uski, An algorithm for the computation of eigenvalues, spectral zeta functions and zeta-determinants on hyperbolic surfaces. *Commun. Math. Phys.* **317**, 827–869 (2013).
32. F. Hecht, New development in freefem++. *J. Numer. Math.* **20**, 251–266 (2012).
33. J. Cook, "Properties of eigenvalues on Riemann surfaces with large symmetry groups," thesis, Loughborough University (2018).
34. J. von Neumann, E. P. Wigner, Über das Verhalten von Eigenwerten bei adiabatischen Prozessen. *Z. Phys.* **30**, 467–470 (1929).
35. C. Fang, H. Weng, X. Dai, Z. Fang, Topological nodal line semimetals. *Chin. Phys. B* **25**, 117106 (2016).
36. R. Donagi, Spectral covers, in *Current Topics in Complex Algebraic Geometry (Berkeley, CA, 1992/93)*, Math. Sci. Res. Inst. Publ., Vol. 28 (Cambridge Univ. Press, 1995), pp. 65–86.
37. A. Selberg, Harmonic analysis and discontinuous groups in weakly symmetric Riemannian spaces with applications to Dirichlet series. *J. Indian Math. Soc.* **20**, 47–87 (1956).
38. R. Laza, M. Schütt, N. Yui, *Arithmetic and Geometry of K3 Surfaces and Calabi-Yau Threefolds* (Springer, 2013).

39. P. Deligne, J. S. Milne, A. Ogus, K.-Y. Shih, *Hodge Cycles, Motives, and Shimura Varieties* (Springer-Verlag, 1982).
40. K. Hori, S. Katz, A. Klemm, R. Pandharipande, R. P. Thomas, C. Vafa, R. Vakil, E. Zaslow, *Mirror Symmetry* (American Mathematical Society, 2003).
41. W. P. Thurston, Three dimensional manifolds, Kleinian groups and hyperbolic geometry. *Bull. Am. Math. Soc.* **6**, 357–381 (1982).
42. S. K. Donaldson, R. P. Thomas, Gauge theory in higher dimensions, in *The Geometric Universe (Oxford, 1996)* (Oxford Univ. Press, 1998), pp. 31–47.3000
43. O. Bolza, On binary sextics with linear transformations into themselves. *Am. J. Math.* **10**, 47–70 (1887).
44. P. Deligne, D. Mumford, The irreducibility of the space of curves of given genus. *Publ. Math. Inst. Hautes Études Sci.* **36**, 75–109 (1969).
45. A.-C. Hladky-Hennion, J.-N. Decarpigny, Analysis of the scattering of a plane acoustic wave by a doubly periodic structure using the finite element method: Application to Alberich anechoic coatings, *J. Acoust. Soc. Am.* **90**, 3356–3367 (1991).
46. P. Langlet, A.-C. Hladky-Hennion, J.-N. Decarpigny, Analysis of the propagation of plane acoustic waves in passive periodic materials using the finite element method, *J. Acoust. Soc. Am.* **98**, 2792–2800 (1995).
47. S. Ballandras, M. Wilm, P.-F. Edoa, A. Soufyane, V. Laude, W. Steichen, R. Lardat, Finite-element analysis of periodic piezoelectric transducers, *J. Appl. Phys.* **93**, 702–711 (2003).
48. R. L. Liboff, *Primer for Point and Space Groups* (Springer-Verlag, 2004).
49. J. F. Cariñena, M. F. Rañada, M. Santander, The quantum harmonic oscillator on the sphere and the hyperbolic plane. *Ann. Phys.* **322**, 2249–2278 (2007).
